# Fat storage-inducing transmembrane (FIT or FITM) proteins are related to lipid phosphatase/phosphotransferase enzymes

**DOI:** 10.15698/mic2018.02.614

**Published:** 2017-12-28

**Authors:** Matthew Hayes, Vineet Choudhary, Namrata Ojha, John JH Shin, Gil-Soo Han, George M. Carman, Christopher JR Loewen, William A Prinz, Timothy Levine

**Affiliations:** 1 University College London Institute of Ophthalmology. 11-43 Bath Street, London, EC1V 9EL, UK.; 2 Laboratory of Cell and Molecular Biology, National Institute of Diabetes and Digestive and Kidney Diseases, National Institutes of Health, Bethesda, MD 20892, USA.; 3 Department of Cellular and Physiological Sciences, Life Sciences Institute, University of British Columbia, Vancouver, British Columbia, Canada.; 4 Department of Food Science and Rutgers Center for Lipid Research, Rutgers University, New Brunswick, New Jersey 08901, USA.

**Keywords:** endoplasmic reticulum retention motif, endoplasmic reticulum stress, lipid biosynthesis enzyme, lipid droplet, remote homology search, type 2 diabetes

## Abstract

Fat storage-inducing transmembrane (FIT or FITM) proteins have been implicated in the partitioning of triacylglycerol to lipid droplets and the budding of lipid droplets from the ER. At the molecular level, the sole relevant interaction is that FITMs directly bind to triacyglycerol and diacylglycerol, but how they function at the molecular level is not known. *Saccharomyces cerevisiae* has two FITM homologues: Scs3p and Yft2p. Scs3p was initially identified because deletion leads to inositol auxotrophy, with an unusual sensitivity to addition of choline. This strongly suggests a role for Scs3p in phospholipid biosynthesis. Looking at the FITM family as widely as possible, we found that FITMs are widespread throughout eukaryotes, indicating presence in the last eukaryotic common ancestor. Protein alignments also showed that FITM sequences contain the active site of lipid phosphatase/phosphotransferase (LPT) enzymes. This large family transfers phosphate-containing headgroups either between lipids or in exchange for water. We confirmed the prediction that FITMs are related to LPTs by showing that single amino-acid substitutions in the presumptive catalytic site prevented their ability to rescue growth of the mutants on low inositol/high choline media when over-expressed. The substitutions also prevented rescue of other phenotypes associated with loss of FITM in yeast, including mistargeting of Opi1p, defective ER morphology, and aberrant lipid droplet budding. These results suggest that Scs3p, Yft2p and FITMs in general are LPT enzymes involved in an as yet unknown critical step in phospholipid metabolism.

## INTRODUCTION

All eukaryotic cells store the neutral lipids triacylglycerol and sterol ester in lipid droplets 1. Understanding this is societally important both for feeding the growing human population 2 and conversely to address problems of the consumption of excess calories with associated metabolic syndrome, type 2 diabetes and atherosclerosis 3. One of the transcriptional regulators of triacylglycerol homeostasis is the peroxisome proliferator-activated receptor-α (PPARα) nuclear hormone receptor, activation of which by fibrate drugs may be beneficial for some atherosclerosis patients 4. The FITM1/2 (Fat storage-Inducing Transmembrane) proteins, also called FIT1/2 (Fat-inducing transcript), are integral ER (endoplasmic reticulum) proteins first identified transcriptional targets of fibrate 5. FITM1 is mainly restricted to skeletal muscle, whereas FITM2 is expressed in most tissues, especially in adipose tissue. They are required for triacylglycerol storage in lipid droplets 5. In support of their biological significance, a SNP (single nucleotide polymorphism) in the FITM2 (C20orf142) promoter is a high risk locus for type 2 diabetes in Asian populations 6,7,8. Homozygous FITM2 mutations introducing a nonsense codon very near to the first residue cause deafness and dystonia 9. This clinical picture is at odds with the effect of homozygous deletion of FITM2 in mice, which causes problems mainly through lack of lipid droplet formation 10,11.

In the short-term, over-expression of FITM does not increase the rate of triacylglycerol synthesis, nor does it increase the expression of genes associated with triacylglycerol synthesis, nor does it reduce the rate of neutral lipid turnover 5. This has suggested that FITMs directly act on the partitioning of triacylglcerol into the storage pathway, possibly affecting its distribution within the ER or its transport from ER to lipid droplets 11,12. In support of this, FITM2 binds directly to both triacylglycerol and its immediate precursor diacylglycerol 12. Also, mutation of a conserved hydrophobic motif in one of the transmembrane domains of FITM2 increases binding to these lipids and increases lipid droplet size 12,13, suggesting that FITM over-activation leads to increased neutral lipid storage. However, both the motif and the lipid binding site are predicted to lie within the membrane, so FITMs are unlikely to act like lipid transfer proteins that transport neutral lipid between organelles 14.

FITM homologues (more closely related to FITM2 than FITM1) have been described so far in animals and fungi, with budding yeast *Saccharomyces cerevisiae* having two FITMs homologues called Scs3p (or FIT2b) and Yft2p (FIT2a). Budding yeast is a useful model for many aspects of neutral lipid metabolism 15,16,17,18, and it may also model pathogenic fungi, since deletion of *SCS3* in the pathogenic fungus *Candida parapsilosis *reduced its virulence 19. Yft2p is often overlooked and its deletion has few phenotypes 19. In contrast, Scs3p is better studied. *SCS3* is required for normal phospholipid synthesis, being one of over 200 genes deletion of which cause inositol auxotrophy, meaning that growth is reduced without the addition of extra inositol, the phospholipid headgroup. On most growth media *scs3*∆ cells have a weak inositol auxotrophy 20 that is rescued by human FITM2 21. However, *scs3*∆ cells have a strong inositol auxotrophy when grown in excess choline 22. The severity of the effect of choline on inositol requirement is almost unique, indicating that Scs3p has a specific molecular function 23. System-wide genetic analysis of yeast lacking both FITM homologues showed links to many pathways including ER stress 21. A strong negative synthetic interaction between *scs3*∆ and deletion of the yeast atlastin (*sey1*∆) 24 suggests that Scs3p promotes homotypic fusion of ER tubules 25. Previously we found that FITMs are required for correct budding of nascent lipid droplets. They first form as neutral lipid lenses up to 50 nm diameter within the leaflet of the ER, and normally bud outwards into the cytoplasm. Without FITMs, lipid lenses fail to emerge from the ER lumen 26.

This evidence suggests that FITMs act in the ER to affect both that organelle and lipid droplets. However, their action at a molecular level is still not known. Here, to gain insight into FITM function, we look at their sequences, where the only published observation is that they have an unusually high proportion of tryptophan residues 27. We identify multiple sequence motifs in FITMs, the most important of which are the catalytic triad of the large family of lipid phosphotransferase (LPT) enzymes. We then show that key residues in this triad are important for FITM function, suggesting that the sequence homology between FITMs and LPTs is physiologically relevant.

## RESULTS 

### Deletion of *SCS3* relocalises Opi1p to lipid droplets

**Figure 1:**
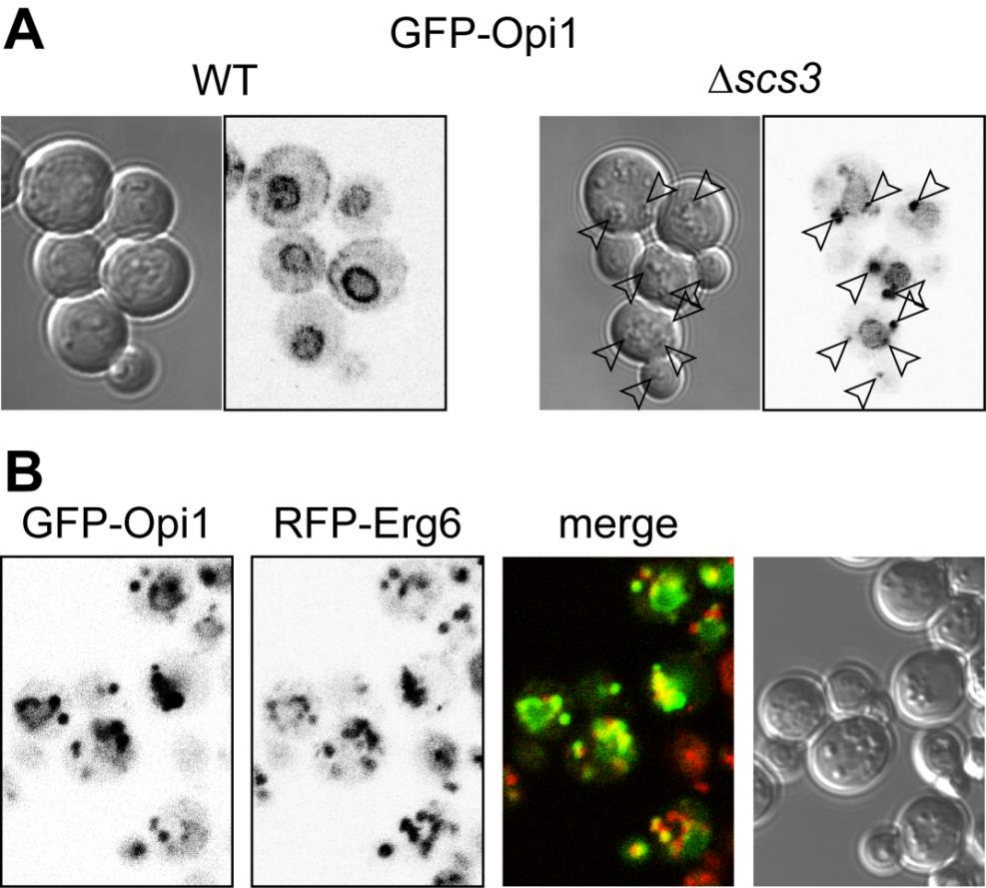
FIGURE 1: GFP-Opi1p re-localises to lipid droplets in cells lacking Scs3p. **(A)** GFP-Opi1p was expressed in wild-type cells (left) and *scs3*∆ cells (right) that were grown without inositol. In wild-type cells, GFP-Opi1p localises most prominently to the nuclear envelope, and also to the cortical ER that forms patches adjacent to the cell periphery 30. In *scs3*∆ cells, GFP-Opi1p is only faintly on the nuclear envelope, and mostly in puncta. Arrowheads indicate these puncta and the corresponding positions in the accompanying transmission image. **(B)** *scs3*∆ cells expressing GFP-Opi1p as in A (1^st^ panel), and co-expressing RFP-Erg6p, a lipid droplet marker (2^nd^ panel), with pseudo-coloured green and red merge (panel 3, superposition = yellow) and transmission image (panel 4).

The rate-limiting enzyme in inositol production by yeast is Ino1p, which is transcriptionally repressed by Opi1p in response to the presence of exogenous inositol, and de-repressed in the absence of inositol 28. With de-repression, Opi1p localises to the ER by simultaneous detection of both the ER protein VAP (called Scs2p in yeast) 29 and phosphatidic acid 30. Since the inositol auxotrophy of *scs3*∆ cells is rescued by deletion of *OPI1*, it has been suggested that Opi1p translocates to the nucleus in *scs3*∆ cells 21. We tested this suggestion, and found that in *scs3*∆ cells grown without exogenous inositol GFP-Opi1p was not seen in the cortical (non-nuclear) ER, but instead it was found on multiple puncta (Figure 1A). By light microscopy these puncta co-localised with bodies that were highly refractile by differential interference contrast (DIC), a characteristic of lipid droplets. To study this further, we co-expressed GFP-Opi1p with the lipid droplet marker Erg6-RFP, and found strong co-localisation when this was done in a *scs3*∆ background (Figure 1B). After addition of inositol, GFP-Opi1p translocated from lipid droplets to the nucleus as in wild-type cells (data not shown). Although the GFP-Opi1p puncta in *scs3*∆ cells are bright, the overall amount of fluorescence per cell is not significantly higher than in wild-type cells (data not shown). As for the nuclear-cytoplasmic distribution of Opi1p, intra-nuclear GFP-Opi1p fluorescence in *scs3*∆ cells was double that of wild-type cells as assessed from fluorescence images (data not shown), which is consistent with the previous work showing Opi1p activation in this strain 21. We noted that deletion of FITMs did not affect the overall number and size of lipid droplets in budding yeast, in agreement with other work 21.

### Deletion of FITM proteins in yeast leads to ER tangles

**Figure 2:**
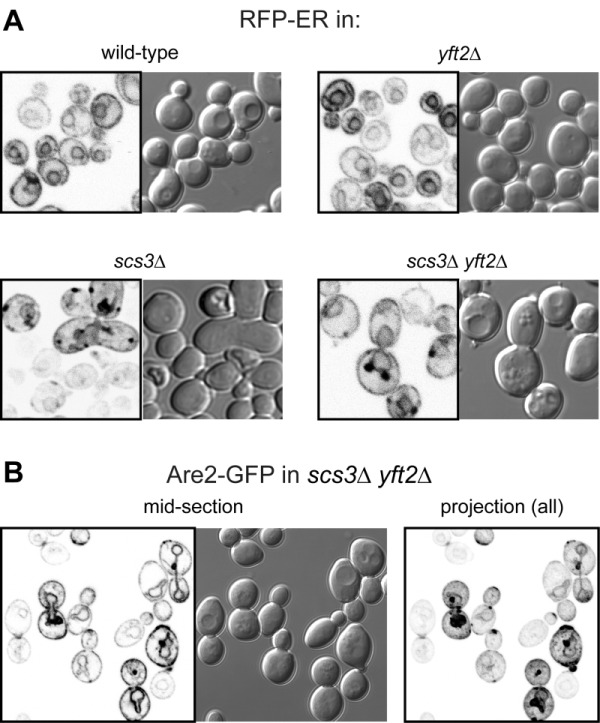
FIGURE 2: Punctate accumulations of ER form in cells lacking *SCS3.* Fluorescent (inverted gray scale) and DIC images of cells with indicated genotype expressing either **(A)** RFP-ER 35 or **(B)** Are2-GFP 36. For Are2-GFP only, a maximum intensity projection generated in Image-J (NIH) from a Z-stack of 17 images 380 nm apart is also shown (right-hand panel).

**Figure 3:**
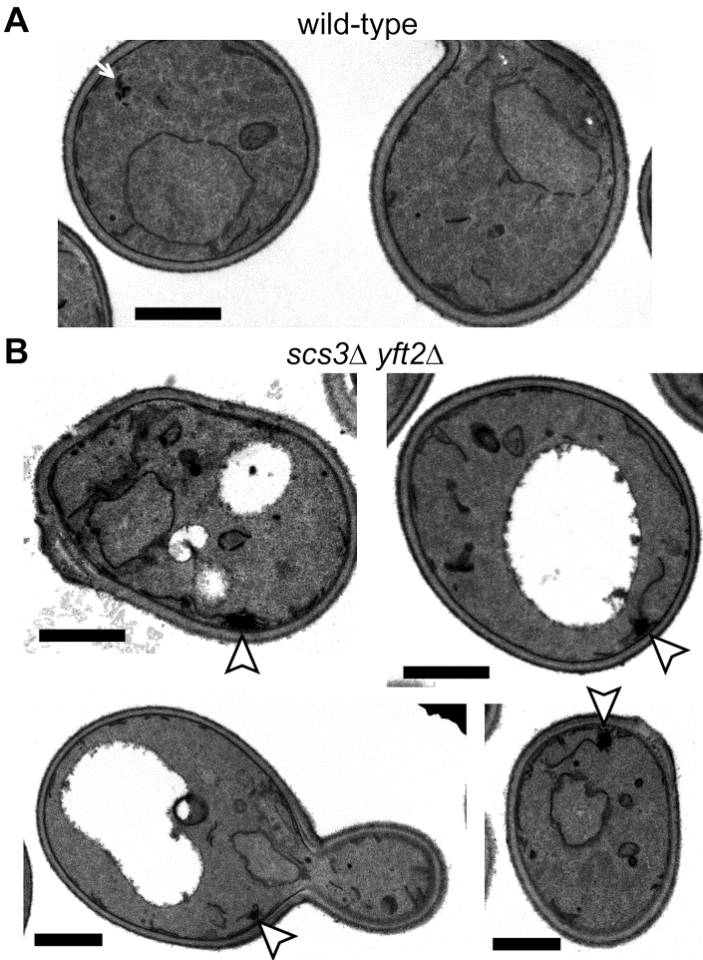
FIGURE 3: Ultrastructure of double delete *scs3*∆ *yft2*∆ cells. Transmission EM images of (A) wild-type cell and (B) a gallery of *scs3*∆ *yft2*∆ cells. In the latter there are tangles of membrane (arrowheads in B), which are absent from the former, although these occasionally have very small stacked membranes (arrow in A). Size bars are all 1 µm.

The close proximity of Opi1-positive puncta to lipid droplets in *scs3*∆ cells is similar to the effect on Opi1p of other mutations affecting phospholipid and neutral lipid metabolism 31,32,33. Since such differences in lipid metabolism can accompany changes in ER structure 15, we compared the structure of the yeast ER in wildtype, *scs3*∆, *yft2*∆ and double delete *scs3*Δ* yft2*∆ cells. In *scs3*∆ and particularly in the double deletion there were punctate structures within the ER, as seen with two different markers, which did not align with lipid droplets (Figure 2A and B). In the *scs3*Δ* yft2*∆ double deletion there were also extended bulges of the nuclear envelope (Figure 2B). To determine the effect of lack of FITM on the ER in more detail, we examined *scs3*Δ* yft2*∆ cells by electron microscopy (Figure 3). This revealed tangles of membrane associated with the ER in 13% of cell profiles (27/214 from 33 consecutive random images) that were hardly ever seen in wild-type cells (1/127 cell profiles, 24 images). Both strains also contain partly stacked membranes that is likely to be the Golgi apparatus 34. The electron micrographs do not identify the origin of the tangles with certainty, but they are likely to correlate with the additional ER-positive puncta.

### Yft2p is found in the endocytic pathway, only being partly retained in the ER

**Figure 4:**
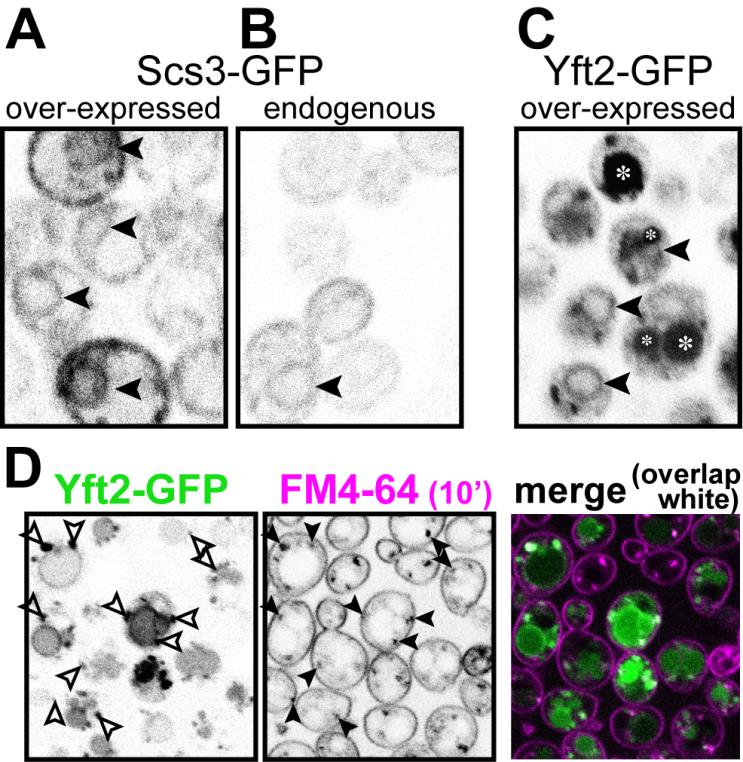
FIGURE 4: Yft2-GFP mainly escapes the ER to reach the vacuole. **(A)** Scs3-GFP expressed from the *PHO5* promoter. **(B)** The same construct expressed from the *SCS3* promoter. Scs3-GFP localises to the ER, with diagnostic nuclear profiles (arrowheads), and only a faint signal with the endogenous promoter. **(C)** Yft2-GFP expressed from the *PHO5* promoter. In addition to some nuclear profiles, there are large spherical regions of diffuse staining (intravacuole - asterisks) and additional puncta. **(D)** Yft2-GFP expressing cells were incubated with FM4-64 on ice to label the plasma membrane, then warmed to 30°C for 10 minutes to allow dye to enter the endocytic pathway, but not to reach vacuoles. Puncta positive for Yft2-GFP (left hand panel) that were also positive for FM4-64 (middle panel) are indicated by arrowheads, and the degree of overlap is also shown in a merged coloured image (right hand panel, GFP = green, FM4-64 = magenta, overlap = white).

In the evolution of FITM proteins, two independent gene duplications are well documented, one producing FITM1 and FITM2 in vertebrates, the other duplication in saccharomycete fungi, the class that includes both *S. cerevisiae* and *Candida albicans*, producing Scs3p and Yft2p 5. Despite the breadth of these duplications, and detailed study of the shared and unique genetic interactions of *SCS3* and *YFT2* 21, it is not yet known how any pair of FITM paralogues are integrated. We wondered if there is any specialisation associated with FITM duplication in yeast. One common divergence between paralogues is in their intracellular localisation. Both human FITM2 and FITM1 localise to the ER 5. Here we expressed Scs3p and Yft2p tagged with GFP at the C-terminus, using the constitutive portion of the *PHO5 *promoter, which causes moderate over-expression 37. Scs3-GFP localised to the ER, both at high levels of expression (Figure 4A) and from its own promoter (Figure 4B), a localisation that we and others have reported before 26,38. For Yft2-GFP, under its own promoter no staining was detected (not shown), as it has been reported previously 38, and the lack of detectability was also true when Scs3-GFP was expressed using the *YFT2* promoter (not shown). We therefore expressed Yft2-GFP using the *PHO5* promoter. This revealed a much wider distribution than for any FITM reported previously. Yft2-GFP was present in the ER, as shown by frequent nuclear envelope profiles (Figure 4C). However, there were additional localisations, with diffuse fluorescence inside the lumen of the yeast vacuole (equivalent of lysosome in animal cells), and multiple puncta associated with the vacuole. Similar intravacuolar diffuse GFP was also seen in some cells expressing Yft2-GFP with the *GPD1* promoter (data not shown). The puncta are characteristic of endosomes, which are typically associated with the vacuole, which we confirmed by showing that many of the puncta were accessed rapidly by the endocytosed dye FM4-64 (Figure 4D) 39. Since the GFP tag is predicted to be cytoplasmic, diffuse intravacuolar fluorescence implies delivery of the whole construct into the vacuolar lumen. Crossing of the vacuolar limiting membrane can result from one of two routes: either by typical secretion with vacuolar protein sorting and ESCRT-mediated (endosomal sorting complexes required for transport) inward budding of the vacuolar membrane, or by autophagy (for example ER-phagy). To differentiate between these possibilities, we expressed Yft2-GFP in strains where either the vacuolar protein sorting pathway or autophagy was inactivated. The vacuolar protein sorting pathway was required for intravacuolar targeting of Yft2-GFP, which was held up on the vacuolar limiting membrane in *vps4*∆ cells, consistent with delivery of this membrane protein through the Golgi and endosomes (Figure S1). In contrast, the distribution of Yft2-GFP was unaffected by the *atg1*∆ deletion, which inhibits multiple routes of autophagy, and Scs3-GFP was unaffected by either deletion (Figure S1). This indicates that Yft2-GFP tends to leave the ER and traffic all the way through the secretory pathway to the vacuole, which it enters by ESCRT driven inward vesiculation.

### FITMs commonly have ER retention motifs, but rarely duplicate to produce homologues that lack the motifs, indicating residence outside the ER

Based on the different localisation of Scs3-GFP and Yft2-GFP, we looked for sequence features that distinguish between them that might explain the difference. For integral membrane proteins, ER retrieval from the Golgi in COP-I vesicles results from a combination of ill-defined luminal signals 40 and two types of well-defined cytoplasmic motifs: lysine-based signals at the extreme C-terminus (either KKxx-stop or KxKxx-stop 41), and di-arginine motifs (RR, RxR and RxxR) that can occur anywhere (and in high copy number) in cytoplasmic extensions or loops 42,43. The function of di-arginine motifs may depend on precise position, for example being less active if they are very close to a transmembrane domain (TMD) 40,44,45. Examination of the FITMs in *S. cerevisiae *shows that Scs3p has 6 di-arginine motifs in predicted cytoplasmic loops, while Yft2p has none. The presence across large families of polytopic proteins either of KKxx motifs and their proven variants 46 or of >3 di-arginine motifs per protein seems to reflect ER localization (Table 1A, lines 1-3). Note that where we counted total motifs in large families all di-arginines were included, not just those in cytoplasmic loops or distant from TMDs. Applying this analysis to the fungal FITM families showed that orthologues of Scs3p consistently have more di-arginine motifs (mean=3.7, n=40) than orthologues of Yft2p (mean=1.6, n=52) (Table 1A, lines 6 & 7). Also, KKxx motifs are more common in Scs3p orthologues (4/40) than Yft2p orthologues (0/52). Therefore, simple analysis of the primary structure of fungal FITMs reveals that the split between Scs3p and Yft2p is associated with high and low propensity to be retained in the ER respectively.

Since groups of Yft2p and Scs3p sequences have a dichotomy in primary structure that explains their different localisations, we wondered whether similar dichotomies have arisen in other FITM duplications. For vertebrate FITMs, there is no such difference, with FITM1s having rates of ER retention motifs that are indistinguishable from the whole FITM family (Table 1A, lines 4 & 5). We next looked at other gene duplications of FITM/Scs3p. The literature to date has reported only the two major duplications in vertebrates and saccharomycetes 5. We found three species outside the vertebrate taxon that have more than one FITM2, while their close relatives have just one: two FITM2s in *Branchiostoma floridae*, the most basal extant cephalochordate; three FITM2s in *Limulus polyphemus *(horseshoe crab); and two FITM2s in the mite *Sarcoptes scabiei*. In each of these species, the duplication events occurred more recently than the FITM1/2 duplication (Figure S2). A comparison of each of the duplicated proteins with the FITM2 that is most closely related, i.e. best shows properties of the common antecedent, showed that the predicted location has changed for one or both duplicates (Table 1B). We also found duplicated FITM2s and no FITM1 in the amphipod crustacean *Hyalella*, but here the duplication event is placed prior to many other speciation events, so a single related sequence cannot be identified. Again the pair of FITM2s diverge in predicted ER retention (Table 1C). We conclude that the five newly described FITM2 gene duplications in four non-vertebrate species have all led to divergent localisations for FITMs. This indicates that the divergence between Scs3p and Yft2p is common rather than unique, and that there is wide-spread pressure for having FITMs both inside and outside the ER.

**Table 1:**
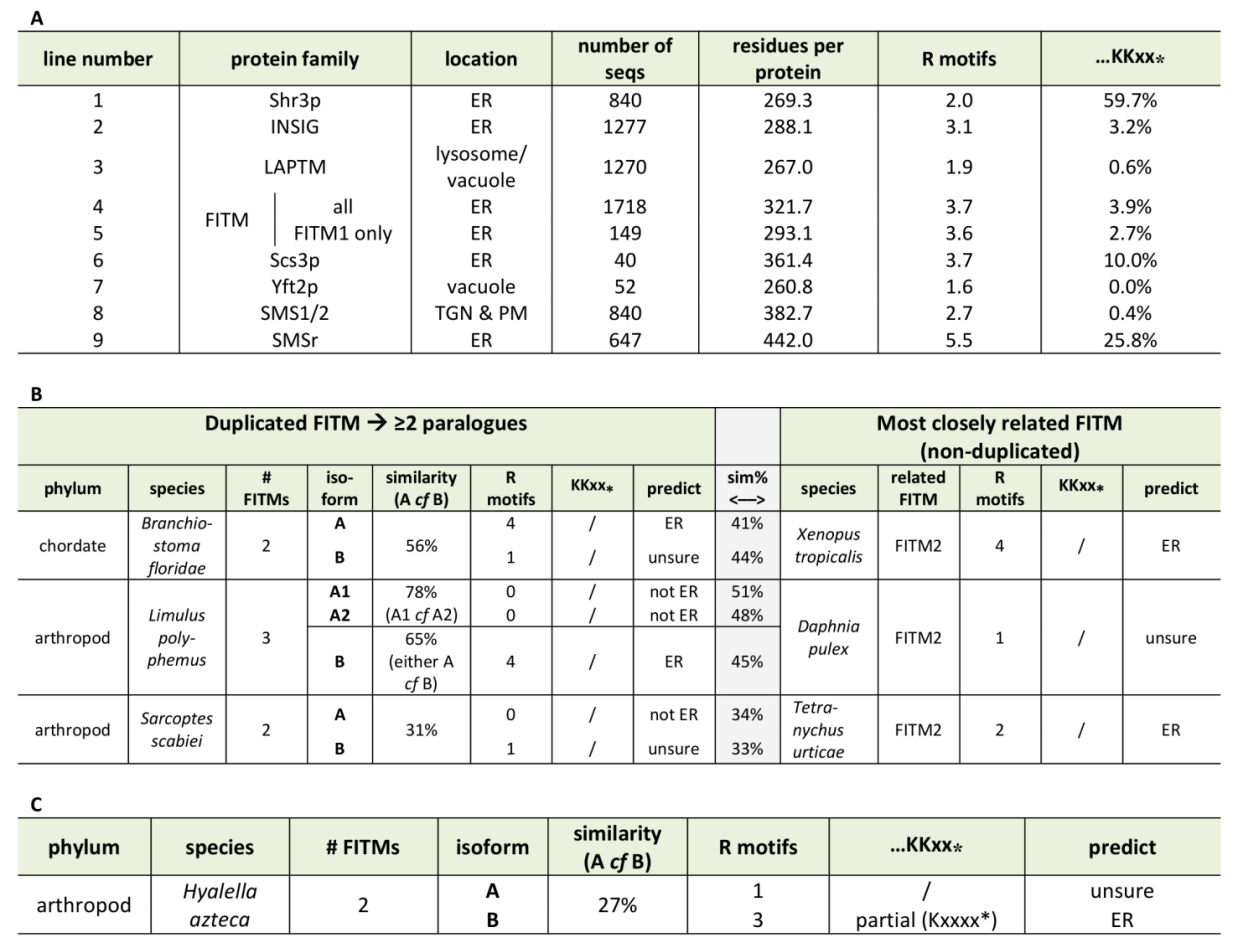
**(A)** ER retention motifs (both di-arginine and C-terminal di-lysine) were compared across multiple large protein families (see Methods). Lines 1-3: examples of ER and lysosomal/vacuolar proteins; 4-7: FITM proteins - all and different subgroups; 8/9: Sphingomyelin synthase subgroups. **(B)** ER retention motifs were used to predict location in three recently duplicated FITM paralogues. Left-hand side: paralogues, including % similarity to each other; right-hand side: the FITM sequence in the most closely related organism, with central shaded column indicating % similarity between sequences on left- and right- hand sides. Prediction of location based on motifs: C-terminal di-lysine or ≥2 di-arginines = ER, 1 di-arginine = unsure, no di-arginines = not ER. **(C)** As B, showing one duplicated FITM pair, where the duplication appears to pre-date many speciation events, making it impossible to identify a single FITM that resembles the presumed antecedent.

### Database mining identifies FITM proteins across the whole of eukaryote evolution

Our mining of the NCBI protein database for species with multiple FITM/Scs3p proteins revealed that several protists (for example, *Toxoplasma*) in the stramenopiles alveolates, and rhizaria (SAR) supergroup have been annotated in an automated fashion as having FITM/Scs3p proteins through the presence of domains in Pfam10261 (Table S1). We generated our own alignments using HHsearch 47 executed as HHpred by the "Tuebingen Toolkit" 48. These searches identified FITM homologues in the protist *Plasmodium falciparum* and in the green alga *Chlamydomonas reinhardtii* (Figure S3). BLAST searches showed that groups of related proteins exist both in protists of the SAR supergroup and in several green algae (Supplementary Figures 2 and 3, and see Figure 6). The conservation is distributed along the whole length of the domain (Figure S3). Therefore, data mining and sequence-based searches with HHsearch and BLAST suggest that FITM was present in the last eukaryotic common ancestor, with selective losses since then, for example in higher plants, which is a wider distribution for FITM than previously appreciated 49.

### FITM proteins have the key sequence LPT enzymes

**Figure 5:**
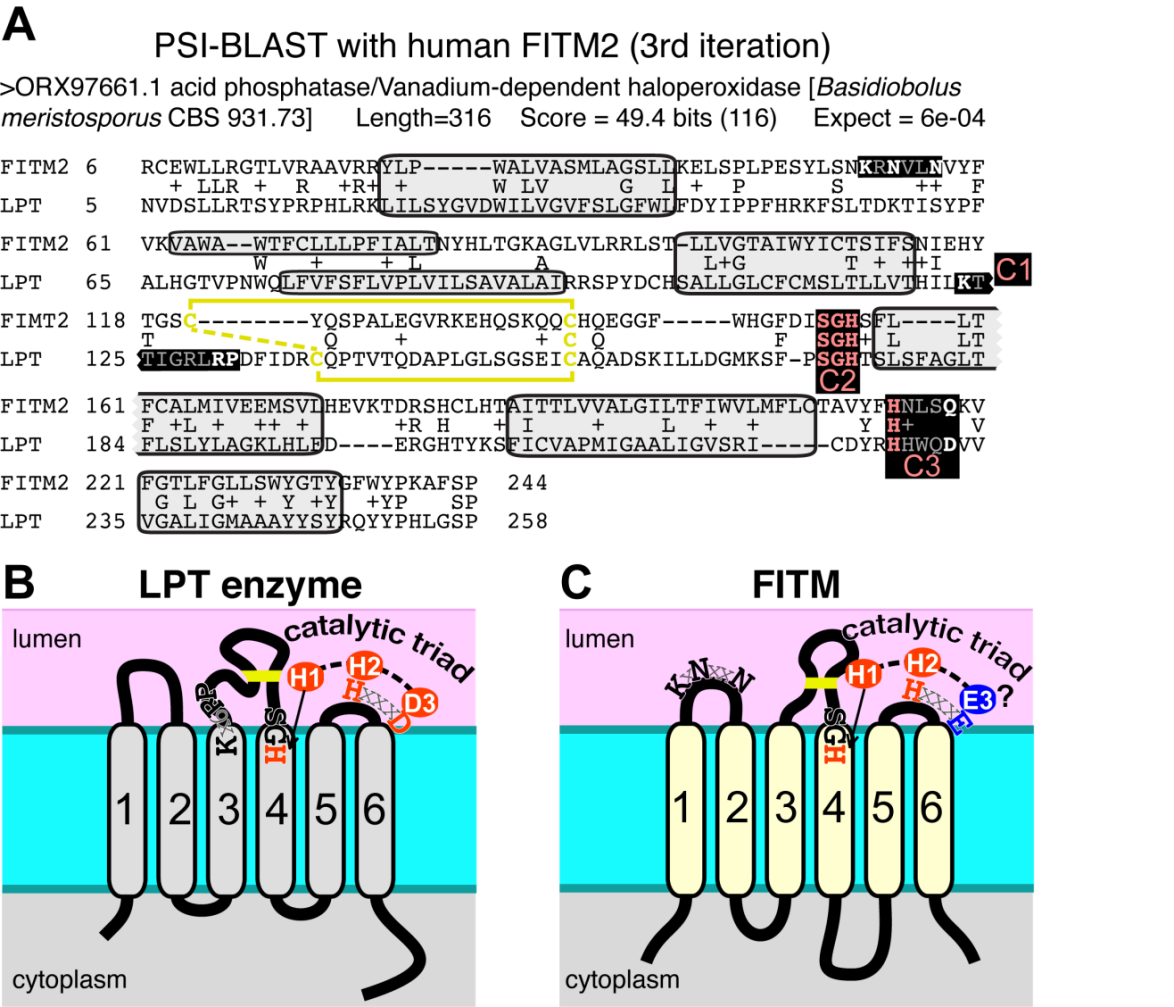
FIGURE 5: FITM proteins are related to lipid phosphatase/phosphotransferase (LPT) enzymes. **(A)** The final significant hit (number 402) from PSI-BLAST with FITM2 after three iterations using the "nr50" database is a fungal LPT annotated as an acid phosphatase. The conserved residues of LPTs occur in three conserved motifs (C1 C2 C3) that are highlighted and colour coded: pink where precisely shared with FITM2 (C2: **SGH**, C3: **H**xxxD/E), white where not shared (C1: **K**-x6-**RP** in LPT only; **K**x**N**xx**N** in FITM2 only; C3: Hxxx**D/E**). Variable positions within motifs are shown in gray. Predicted transmembrane domains (TMDs, shaded in light gray) align well in five cases. Paired cysteines likely to form a disulphide bridge in the loop between TMD3 and TMD4 are shown in yellow. **(B)** Topology of a typical LPT enzyme, Dpp1p in yeast, with the three conserved motifs, within which the catalytic triad of the most invariant residues are shown in red. **(C)** Topology of human FITM2, with the two motifs shared with LPTs and the KxNxxN motif. Residues that align with the catalytic triad are coloured red where identical with LPTs, and blue where non-identical.

Since database searches were informative on FITM evolution, we wondered if they would also provide information on the function of FITMs. We therefore looked for proteins with functional annotations that align with FITM2 in sensitive alignments with PSI-BLAST 50. The alignment to human FITM2 using the full protein database was dominated by >1000 highly related sequences. Therefore, we repeated the search using a non-redundant-50 (nr50) protein database, where groups of sequences >50% identical are reduced to single entries 51. From the third iteration onwards, this produced significant hits to LPT enzymes (Table S2A). These are integral membrane enzymes in the type 2 phosphatidic acid phosphatase (PAP2, Pfam01569) family, which also includes soluble vanadium-dependent chloroperoxidases in prokaryotes 52. With further iterations, LPTs became increasingly common in the alignments (Table S2B). Searches into the complete database (i.e. nothing excluded because of high levels of sequence identity) identified a similar range of LPTs, but the statistical dominance by genomes related to key model organisms (mammals, saccharomycetes) caused alignments with LPTs to be non-significant (data not shown). The FITM-LPT alignment was strongly supported by HHpred, the closest hit being the LPT enzyme Dpp1p, with probability of shared structure = 97% across 207 residues (data not shown). A key aspect of the alignment between FITMs and LPTs is that the residues conserved across the alignment include the LPT active site (Figure 5A) 52. Although this does not distinguish between convergent and divergent evolution, it indicates that FITMs are functionally related to LPTs.

**Figure 6:**
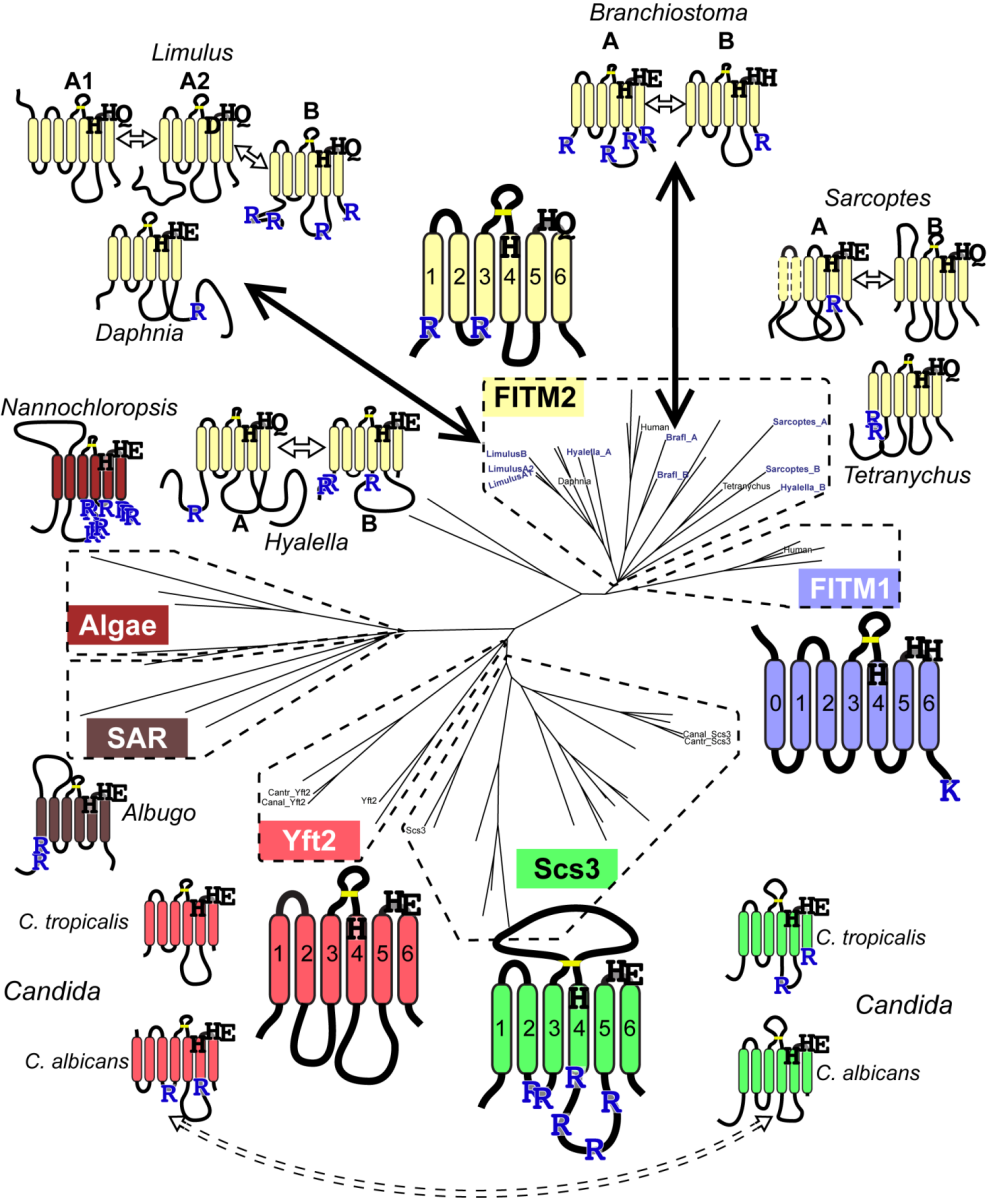
FIGURE 6: Catalytic triad residues and di-arginine motifs diverge at multiple duplications across FITM evolution. Sequence features of main FITMs and those in recent divergent duplications. Examples from the major groups of FITMs (FITM1 in vertebrates, FITM2 in animals, Yft2p and Scs3p in fungi) and from the two groups of FITMs in protists and algae are shown to indicate the residues that align with the catalytic triad in LPTs and their ER retention motifs (R = diarginine, K = KK(x)xx). Also shown are the recently duplicated sequences and their closest related sequences, as described in the text, colour coded to indicate what group of FITMs they belong to. Other features indicated: size of loops between TMDs, disulphide bridge predicted for loop 3/4 (yellow bar), and the position of each protein on the overall tree of FITMs (see Figure S2). Sequences from *Candida* species *C. tropicalis* and *C. albicans* show high levels of conservation, but the di-arginine motifs are swapped (see Discussion).

Which structural features of LPT enzymes are also found in FITM proteins? The LPT family consists of polytopic proteins, typically with 6 TMDs (range 4-8) (Figure 5B) 52. The catalytic centre of LPTs is always situated in the reduc-ing environment of the lumen of the secretory pathway or the external milieu, and the active site consists of three motifs called C1, C2 and C3 53. C1 is at the N-terminus of the long intra-lumenal loop 3/4, which is structured by a disulphide bond, C2 is at the C-terminus of the same loop, and C3 is in the adjacent short loop 5/6 (Figure 5B) 53. The catalytic triad for LPTs consists of two histidines and one aspartate (here called H-1, H-2, D-3), where the C2 motif contains H-1 typically as "SGH", and the C3 motif contains H-2 and D-3 precisely spaced HxxxD 52. Most of these features are found in FITM proteins. They typically have 6 TMDs (Figure 5C) 13, although human FITM1 is predicted to have an extra TMD at its N-terminus (Figure 6). FITMs also have many of the critical elements of the LPT enzymatic centre, including SGH in C2 and a disulphide bond in loop 3/4. However, C3 varies from H-x-x-x-D, as the fifth residue in that sequence is typically glutamate (making H-x-x-x-E). Sometimes this position is glutamine (Q) or H, and rarely D (Table S2C), so that the overall range of residues is similar to LPTs though in different ratios (Table S2D). Finally, C1 which contains K-x6-RP in LPTs is missing from FITMs, but the first main conserved block of FITM sequence (Figure S4) shows that the luminal loop 1/2 contains a conserved "[K]xNØØN" motif, where [K] indicates the predominance of K at that position and Ø is a hydrophobic residue (FILV) (Figure 5C).

We next looked for other primary structural features that specifically relate to possible homology between FITMs and LPT-like enzymes. Firstly, we looked to see if the divergence of ER retention motifs also exists in any group of LPTs that has undergone duplication and divergence. This applies to sphingomyelin synthases (SMS), which have two divergent groups: the active enzymes (SMS1/2) localised in the late secretory pathway 54, and the SMS-related proteins (SMSr) that localise to the ER and act as lipid sensors 55. SMSr proteins have more of both di-arginine motifs and KKxx motifs than SMS1/2 (Table 1A, lines 8/9), showing a difference in easily discernible motifs that is similar to the difference between Scs3p and Yft2p, although there are more di-arginine motifs in SMS proteins than FITMs. Secondly, we examined if there was any link between predicted cellular location and the residues in the predicted catalytic active site. Of the seven duplications (2 major and 5 minor), five are associated with divergence of a catalytic residue, including FITM1 *vs.* FITM2 (Figure 6). For *Limulus* where there have been two duplications, the split between FITM2A and FITM2B led to divergence of localisation but not in catalytic residues. By comparison the split between FITM2A1 and FITM2A2 has created in the latter a unique variant (H-1-D) that is likely to inactivate any LPT-like activity. The only duplication with completely conserved catalytic residues is the major one across saccharomycete fungi creating Yft2p and Scs3p. However, here there is variation in the conserved [K]xNØØN motif in loop 1/2, which is present in Yft2s (48/53), but is largely absent from homologues of Scs3p (13/40, Figure S5). Thus, the duplications within this family affect not only localisation, but also the likely architecture of the site related to the LPT catalytic centre.

Overall, many features are shared by FITMs and LPTs, indicating that the FITM family has evolved from the LPTs, and should be included in the overarching LPT family. However, the substitution at D-3 for E-3 calls into question whether FITMs are catalytically active 55. In addition, the alignment provides no information on possible substrates.

### The conserved residues aligning with the catalytic centre of LPTs are functionally important

**Figure 7:**
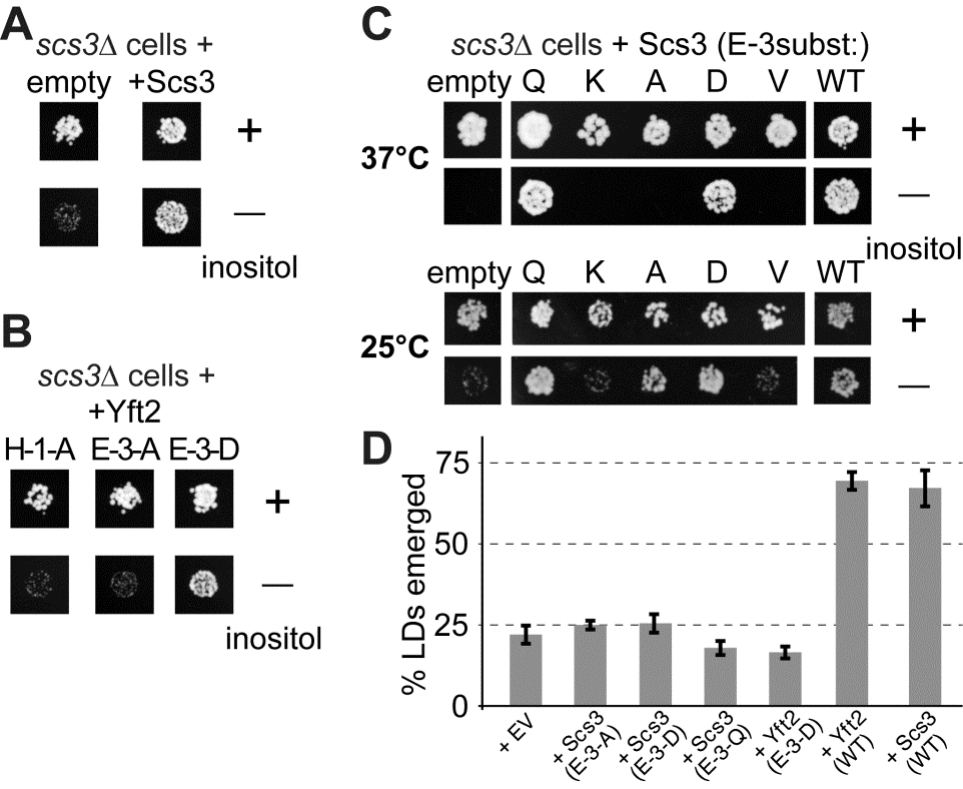
FIGURE 7: Growth of yeast lacking inositol requires FITMs with an intact catalytic triad. **(A-C)** Equal numbers of *scs3*∆ yeast (~50) transformed with the plasmids indicated were spotted onto plates either with added inositol (100 µM) or without, and grown at 30°C or the temperature indicated for 48-72 hours. **(A)** Cells either transformed with empty plasmid (expressing GFP alone) or expressing Scs3-GFP. **(B)** Cells grown in parallel to A, but expressing Yft2-GFP, either mutated H-1-A, E-3-A or E-3-D. **(C)** Cells were expressed with mutants of Scs3-GFP, substituting E-3 to five other residues. Here cells were grown at high stringency (37°C) or low stringency (25°C), the latter allowing rescue by the partially active construct E-3-A. **(D)** Emerged lipid droplets were counted in random sections of *scs3*∆ *yft2*∆ yeast expressing the indicated constructs (EV = empty vector), and identified as emerged from the ER by the complete absence of wrapping membrane (≥50 cells each experiment, n=3, SD shown by error bars). Emergence, as opposed to remaining wrapped in ER, was scored as previously 26. This means that the droplets not counted here (*i.e. *not emerged) include both the clearly wrapped, and those with an ambiguous morphology (~7% under all conditions).

The *SCS3* gene was originally identified through a screen for deletants showing inositol auxotrophy, particularly in the presence of choline 22. In *scs3*∆ cells inositol supplementation is required more at raised temperature (37°C compared to the usual 30°C) 23. *yft2*∆ has no significant inositol phenotype, and the double delete *scs3*∆ *yft2*∆ has the same inositol auxotrophy as *scs3*∆ alone 21. Re-expression of Scs3p on over-expressing plasmids where the open-reading frame is followed by GFP completely rescued the ability of a *scs3*∆ mutant to grow on inositol-deficient medium (Figure 7A). To test the functional relevance of the alignment of FITM with LPTs, we generated constructs mutated in key enzymatic residues. The role of H-1 was tested in Yft2p, which when over-expressed from the *PHO5* promoter rescued the inositol auxotrophy of *scs3*∆ cells. Substitution of H-1 with alanine (A) abolished the ability of over-expressed Yft2p to rescue inositol auxotrophy (Figure 7B, left-hand panel). We also tested the role of E-3 in some detail. In Yft2p, the substitution E-3-D had no effect, while E-3-A almost completely inhibited growth (Figure 7B). For Scs3p we made a wide range of substitutions of E3. The conservative changes E-3-D and E-3-Q had little effect on activity, while substitution with highly variant residues, either lysine (E-3-K) or valine (E-3-V), abolished activity completely, so that growth was as weak as with an empty plasmid (Figure 7C). By comparison, the neutral substitution E-3-A inhibited growth at 37°C, a temperature for which yeast have a high stringency for Scs3p function 23, but under less stringent conditions (25°C) the E-3-A mutant supported growth (Figure 7C). This indicates that Scs3p with the E-3-A substitution is partially active for supporting growth without inositol.

Alongside the inositol auxotrophy assays, we also determined the expression and localisation of the Scs3p mutant constructs, and found that they are all expressed at similar levels in the ER (Figure S6A/B). This also showed which variants of E3 rescue the formation of ER tangles: E-3-Q and E-3-D rescued like wild-type, but E-3A/V/K did not (Figure S6B). In addition, we studied the ability of plasmid borne Scs3p to rescue GFP-Opi1p relocalization to lipid droplets. Here we found a spectrum of activity where E-3-Q (almost as active as wildtype) was clearly different from E-3-D (only partly active), and E-3-A was inactive (Figure S6C).

Another strong phenotype of yeast lacking both FITMs (*scs3*∆ *yft2*∆) is that lipid droplets fail to bud into the cytoplasm, remaining wrapped in ER membrane, a phenotype that is rescued by both Scs3p and Yft2p 26. Here we found that mutations in E-3 of either protein, even conservative substitutions with Q or D, completely inhibited the rescue of lipid droplet budding (Figure 7E). Overall, these results indicate the necessity of the conserved triad H-1 H-2 E-3 for FITM proteins to function. For each of the four phenotypes we studied, Scs3 with the most conservative substitutions at E-3 tended to retain more activity (activity order Q>D>A>K/V). However, the phenotypes varied considerably in the extent to which any mutation was tolerated: inositol auxotrophy and ER tangles were rescued fully by two mutants (Q and D), GFP-Opi1 mistargeting was largely rescued by one mutant (Q), and lipid droplet budding was not rescued at all, even by the Q mutant.

We also obtained two sets of negative results. We tested whether FITMs are phosphatidic acid phosphatases, an activity commonly associated with LPT enzymes and already known for two of the seven previously identified yeast LPTs (Lpp1p and Dpp1p). We expressed either Scs3p or Yft2p at high levels (*GAL1/10* promoter, cells using galactose as carbon source) in cells lacking Lpp1, Dpp1p and also the other two known phosphatidic acid phosphatases Pah1p and App1p 56. Extracts of these cells showed no significant phosphatidic acid phosphatase activity above background (Scs3p: 0.005 U/mg, StdDev = 0.009, n=6; Yft2p: 0.007 U/mg, SD = 0.016 n=6), i.e. signal 1/1000^th^ of wild-type cells (5.2 U/mg, SD = 0.12, n=3). This indicates that FITMs do not have significant phosphatidic acid phosphatase activity. Also, because of the link between FITM2 mutation and mitochondrial dysfunction in humans 9, we examined mitochondrial morphology by EM in *scs3*Δ* yft2*∆ double-delete cells grown on fermentable sugar to enforce the Krebs cycle, but there was no difference from wild-type (data not shown).

## DISCUSSION

FITM proteins are required to maintain the normal storage of triacylglycerol in many different animal cells. In yeast, this quantity is not affected by FITM deletion 21 instead, a more subtle effect is that FITM proteins are required for correct budding of nascent lipid droplets, a phenotype that is conserved in human cells 26. This altered budding appears to provide a novel mechanism by which FITMs affect neutral lipid metabolism, but it in turn calls for a molecular explanation for the events that lead to LD budding. Work in yeast has identified another phenotype, a choline-sensitive inositol auxotrophy, which indicates a specific role in phospholipid metabolism. The similarity between FITMs and LPTs we have identified here suggests that FITMs interact with specific phospholipids, in addition to the previous identification of direct binding to non-phospho-lipids (di- and tri-acylglycerol) 12.

**Figure 8:**
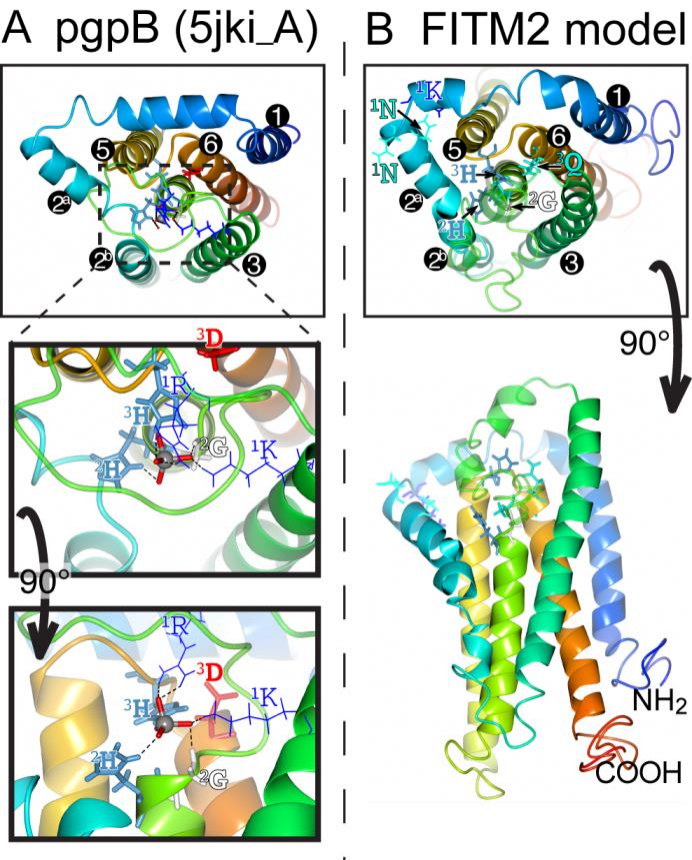
FIGURE 8: Structural model of FITM2. **(A)** Lumenal aspect of bacterial PgpB enzyme (accession 5jki, bottom panel), which is the highest scoring threading template for FITM2 identified by I-TASSER, numbering its 6 TMDs, except the central TMD 4, and with TMD2 split in 2 parts. Zoom in (middle panel) and rotation to side aspect (top panel) show the highlighted elements of the active site, including the tungsten ion (gray sphere) crystallised in place of the phosphate group in 5jki, and the side chains of co-ordinating conserved residues (coloured by residue type) in C1 (1K, 1R = thin bonds), C2 and C3 (2G, 2H, 3H, 3D = fat bonds) 60. **(B)** Superimposed view of the modelled structure of human FITM2 with side-chains of the conserved residues in C2 and C3 as in A (bottom panel = lumenal aspect, top panel = side aspect), plus the hydrophilic residues (KxNxxN) conserved in loop 1/2 (thin bonds). Models were obtained from the I-TASSER server for 3D structure prediction with standard settings 61. Several models with seven TMDs were rejected on the basis of topology studies of Yft2p and Scs3p showing six TMDs 13. In all panels, colouring of the chain graduates from blue (N-terminus) to red (C-terminus).

The main question raised by our study is whether FITMs are active LPT enzymes. The strongest evidence is in the sequences of FITMs dispersed across evolution. The residues FITMs share with LPTs were already identified as among the most conserved in FITMs 13, and form the core of two of the three main blocks of conserved sequence in FITMs. The FITM block that matches LPTs best includes the C2 motif and "SGH" (Figure 5A). Previously these residues have been part of a wider "FITM signature sequence" that includes most of TMD 4. Within this motif, the glycine (G in "SGH") is not universal in FITMs, being replaced by S in FITM1, and D in all SARs and algae. These substitutions are not necessarily inactivating for an LPT enzyme, as sphingosine phosphate phosphatases have threonine at this position 52, dolicholpyrophatases and plant LPTs mainly have serine 57, and the antibiotic hydrolase RfiA from *Arthrobacter* has leucine 58. Our Yft2p H-1-A mutant showed the importance of this residue for inositol auxotrophy. Unlike the C2 motif, FITMs lack the C1 motif of LPTs that helps co-ordinate the phosphate (Figure 8A). However, FITMs have a [K]xNØØN motif in the adjacent lumenal loop 1/2. A structural model of FITM2 based on the structure of PgpB, a bacterial polytopic LPT, shows that this loop is wrongly placed to co-ordinate the phosphate (Figure 8B). Nevertheless, other active LPTs such as SMSs also lack the C1 motif 52, instead having a family-specific motif in loop 1/2 54. We note that a domain of rotaviral VP3 capping enzymes that is also distantly related to LPTs, having SGH and HxxxE/D motifs, also has a NxxN motif 59, so this may have a specific function in phosphotransferase reactions.

This leaves the C3 motif, where the third residue in the catalytic triad tends to be glutamate (E-3) in FITMs rather than aspartate (D-3) in LPTs. Despite the D-3-E substitution abrogating the activity of some LPTs 55, D-3 is not invariant in LPTs (Table S2). For example, in dolichol pyrophosphate phosphatases this residue is always glutamine. We found that variation of E-3 reduced the activity of yeast FITMs in all the phenotypes we tested. Where the catalytic triad has been mutagenized more extensively, for example in PgpB, H-2 is essential for all activity, but variation in either H-1 or D-3 affects substrates variably 62. The natural substrate of PgpB is phosphatidylglycerol phosphate, but it can hydrolyse several other lipid phosphate substrates, which are more sensitive to H 1-A and D 3-A mutations than is the natural ligand. Therefore, the variation in effect we detected for the same mutation in different Scs3p-dependent functions (D and Q permissive for inositol auxotrophy and ER morphology, only Q permissive for Opi1p targeting, neither substitution rescuing lipid droplet budding) may indicate that a different range of substrates can fulfil each function. Increasing the severity of mutation (E(Q<D<A<K/V) reveals that some functions of Scs3p have greater substrate specificity than others. The only reaction we have directly tested is the canonical phosphatidic acid phosphatase reaction, where extracts of yeast over-expressing either FITM failed to hydrolyse phosphatidic acid to diacylglycerol and phosphate. Overall, the conserved motifs and specifically positioned residues in FITMs are consistent with them being LPTs, though we have no direct evidence of such activity.

There are groups of pseudo-enzymes within the LPT family, so next we consider if this is the situation for FITMs. The most well-known LPT pseudo-enzymes are the lipid phosphatase-related (LPR) proteins (also called plasticity-related genes, PRGs). These have roles in the handling of extracellular phospholipids such as lysophosphatidic acid, but rather than catalyse a reaction, they seem to sense the lipid through altered ability to bind signaling partners 63,64. Consistent with this, H-2 in LPR/PRGs is always substituted (N or S) and some other key residues are variably missing, so that phosphatase activity is likely to be abolished 52. In FITMs, such major changes are only found in a few FITM1s, particularly in fish. This suggests that the vast majority of FITMs are active LPTs.

The predicted lack of function in the subset of FITM1s with altered key residues is interesting in relation to the dimorphism we found throughout the FITM family. In addition to the FITM2/FITM1 and Scs3p/Yft2p duplications, we mined data on 5 further duplications. All seven events show divergence in either their catalytic motifs or their ER retention motifs (Figure 6). This indicates that there is evolutionary pressure to provide a second intracellular location and/or spectrum of substrates for FITM. In relation to this, residues adjacent to the core catalytic sites can determine specific substrates 65. For FITMs, the wider definition of C3 is TAØYF**H**TØØ**E**KØØG, with the F^-1^ before HxxxE and K^+5^ after HxxxE being highly conserved. This suggests that the minority of FITMs without these residues will have different specificity (e.g. K^+5^ missing in SAR/algal sequences). FITM models based on crystal structures of PgpB (Figure 8) might be used in future to determine what substrates fit into FITMs.

Regarding the di-arginine motifs we have found in FITMs, these have been identified widely, from humans to plants 40,41,42,43, but they have not been studied in a system-wide fashion and they are not included in any available intracellular localisation algorithm, such as PSORT 66. Application of a crude high-throughput method that did not account for predicted topology (hence counting motifs in the lumen) still discerned between ER residents and others, and it showed a divergence between Scs3s and Yft2s. Such short motifs as a pair of arginines separated by 0, 1 or 2 residues might evolve easily, allowing the numbers to vary considerably between closely related proteins; e.g. horseshoe crab (*Limulus*) FITM2A has zero vs. FITM2B has 4 (Figure 6). A further example of apparent diversification of arginine motifs is found in *Candida albicans.* This saccharomycete has a Scs3p/Yft2p pair closely related to the *C. tropicalis* pair (both >50% identical). However, the di-arginine motifs in Scs3p in *C. tropicalis* and most fungi, are swapped in *C. albicans* into Yft2p (Figure 6, dashed arrow). This suggests that divergence between the intracellular localisation of FITM paralogues could be selected independently of which paralogue goes where.

Our results do not provide the explanation for the link between phospholipid and neutral lipid metabolism that must eventually be found, but they do provide some tools for future work. We have studied the role of Scs3 in multiple phenotypes, all linked to lipid metabolism. Is it possible to use these to identify a likely substrate/product of FITMs? Increased Opi1p targeting at an intracellular site might reflect accumulation of phosphatidic acid 30, but matters are more complex. As well as the lipid headgroup, Opi1p shows a marked preference for phosphatidic acid with short acyl chains 67, and even binds to lipid droplets because of other the features altogether related to lipid packing 31. Thus, none of the phenotypes we studied identify a specific lesion in lipid metabolism.

Given the failure of high-throughput approaches in yeast to report a major lipid imbalance in cells lacking FITMs, we favor the possibility that their function is hard to detect in one of three ways. Firstly, it is possible that FITMs are lipid sensors, similar to SMSr enzymes that use their synthetic activity to make very small amounts of ceramide-phosphoethanolamine in the ER, this small pool having large downstream effects by regulating the much larger ER pool of the ceramide substrate 55. This mysterious mode of action might only be assayed using purified protein, which could be the subject of future work. The second way that the action of FITMs might be hard to detect is if they carry out a phospho-exchange reaction, swapping a phosphate-containing headgroup between two highly similar lipids. Another LPT that does this is phosphatidylcholine:diacylglycerol cholinephosphotransferase (PDCT) in plant seeds, which swaps phosphocholine between two different diacylglycerols 68. The biochemical effect of PDCT might be hard to detect because the reaction is pseudo-symmetrical, with substrates and products being identical in terms of headgroup, hence indistinguishable by thin-layer chromatography, which identifies main lipid classes only. Finally, FITM activity might be hard to detect if the enzyme functions in a confined sub-domain of the ER where lipids cannot freely diffuse, analogous to sub-domains enriched in newly synthesized PC 69. The strong genetic link in yeast to *SEY1*, the atlastin homologue, might be explained by FITM acting specifically where fusions are about to occur within the ER network 25. Highly restricted activity may also occur at sites of lipid droplet budding. Future investigations might determine if rapid alterations in FITM activity has immediate effects on the levels of unique lipid species, as identified by mass spectroscopy, which splits the lipidome into thousands of lipid species 70. A system-wide search for such subtle effects of FITMs may be the key to unlocking their function.

## MATERIALS AND METHODS

### Plasmids

For fluorescence microscopy and inositol auxotrophy assays, Scs3p and Yft2p were expressed from the constitutive portion of the *PHO5* promoter 37 and followed by the linker GTGPVEK then GFP in the plasmid pRS416 (CEN/URA). Constructs were checked by sequencing. Single point mutants were made as follows (and checked by sequencing): Scs3p E-3 (E354) to D, Q, A, K and V; Yft2p H-1 (H178) to A, and E-3 (E243) to D and A. For EM, the same mutations were introduced into plasmids pNO18 and pNO19, which contain the *YFT2* and *SCS3* open-reading frames respectively, with their own promoters in the plasmid YCplac33 (CEN/URA).

### Strains

Wild-type yeast were BY4741, and single deletion strains were obtained from freezer stocks of the yeast deletion collection (BY4741, Mat a, and ∆*open-reading-frame*::KanMX). Double delete yeast (*scs3*∆* yft2*∆) were made from the *yft2*∆ single delete strain by PCR knock-out using the *S. pombe* HIS5 gene, as previously 37. Cells with Erg6 tagged with RFP in the genome were obtained from a collection of strains with marked organelles 71.

### Light microscopy

Yeast growing in log phase were examined with a confocal microscopy system (AOBS SP2; Leica) at room temperature (63× NA 1.4 objective) using LCS software (Leica) for acquisition.

### Electron microscopy

Yeast in mid-log phase were fixed and embedded in Spurr’s resin for transmission EM. For ER structure, fixation was in potassium permanganate 1.5% 35, and images were taken at random from low-power fields with high cell density. For lipid droplet visualisation, fixation was in 1% glutaraldehyde and 1.25% paraformaldehyde 26.

### Bioinformatics

#### Basic motifs

Di-arginine motifs (RR, RxR and RxxR) for individual proteins in Figure 6 etc. were mapped onto the topology predicted by TOPCONS 72, and only cytoplasmic motifs were counted. For the high throughput analysis of motifs in large families in Table 1A, the link between motif and predicted position in the cells was not applied, therefore non-functional motifs (in luminal loops - typically there is one or more in loop 3/4) are included. For C-terminal di-lysine motifs, all instances of KxKxx-stop and KxKxx-stop were included, as well as proven variants RxKxx, KxRxx, HxHxx, KxHxx and RKxx 46.

#### Tree

57 FITM sequences from the organisms in Table 1B and Figure 6 and from representative eukaryotes were aligned by CLUSTAL Omega at EBI 73. The full alignment of the 57 sequences is available as Supplementary File 1. Then, a tree was generated by submitting the alignment to PHYML 3.0 at www.trex.uqam.ca 74, with standard settings and branch support by 100 replicates for bootstrapping.

#### Data mining and alignments

The NCBI protein database was searched with the term "scs3p AND (alveolata OR stramenopiles)". To expand the families of protist and algal FITM homologues, entries in both groups were submitted to standard BLAST at NCBI. To identify distant relatives of FITM proteins, PSI-BLAST was seeded with human FITM2 and used to interrogate the nr50 protein database in the Tuebingen Toolkit for 5 iterations 51. Alignments were viewed in JALVIEW with Clustal X colours. HHpred at the Tuebingen Toolkit 51 was seeded with FITM sequences and used to search for homologues in PFAM and model eukaryotes. For Figure S3, the hit in *Chlamydomonas reinhardtii *was itself used to seed a reverse search at HHpred, with the width of alignment shown expanded (without changing the statistics of significance) by setting the MAC realignment threshold to 0.1 (default = 0.3).

### Phosphatidic acid phosphatase assay

Cell extracts were prepared from the *pah1*∆* app1*∆* dpp1*∆* lpp1*∆ mutant overexpressing Scs3p or Yft2p from the *GAL1/10* promoter. Activity was measured by following the release of water-soluble [32P]Pi from chloroform-soluble [32P]phosphatidic acid as described previously 75. Units are nmol/min and are expressed per mg cell protein.

## SUPPLEMENTAL MATERIAL

Click here for supplemental data file.

All supplemental data for this article are also available online at http://microbialcell.com/researcharticles/fat-storage-inducing-transmembrane-fit-or-fitm-proteins-are-related-to-lipid-phosphatase-phosphotransferase-enzymes/.
