# Supplementary material for: Fat storage-inducing transmembrane (FIT or FITM) proteins are related to lipid phosphatase/phosphotransferase enzymes
Source: Microb Cell. 2017 Dec 28;5(2):88–103. doi: 10.15698/mic2018.02.614 (PMC5798408; doi:10.15698/mic2018.02.614)
Supplement: Supplementary file 1 [file mic-05-088-s01.pdf]

Figure S1

A

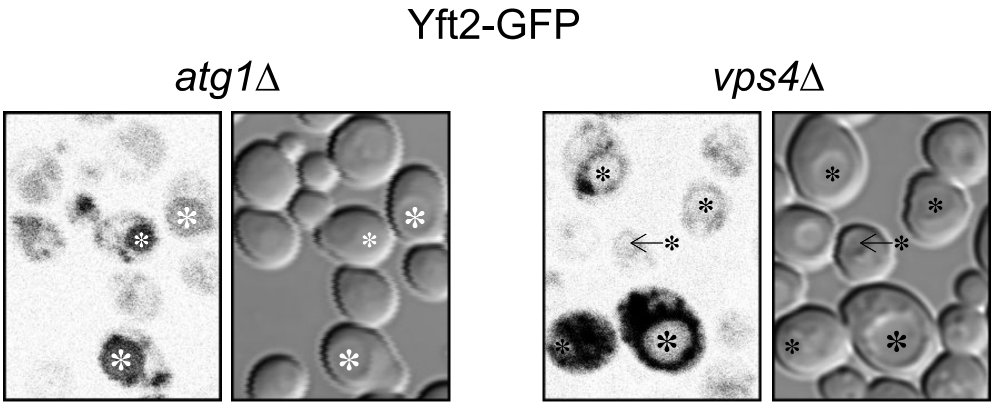

B

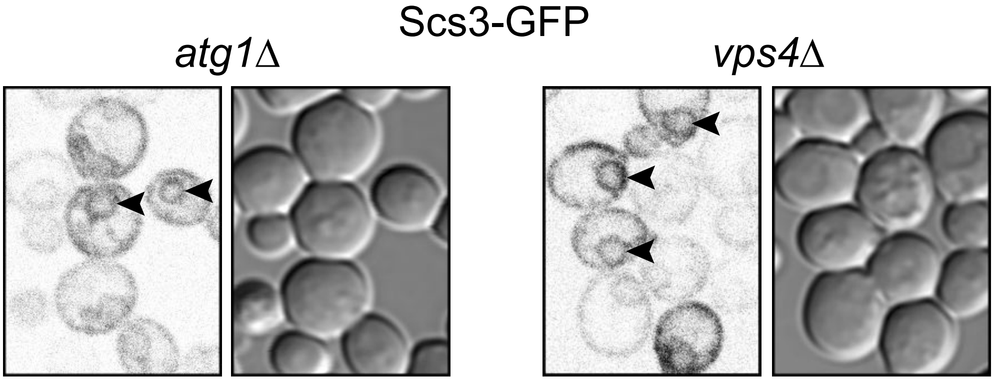

Figure S2

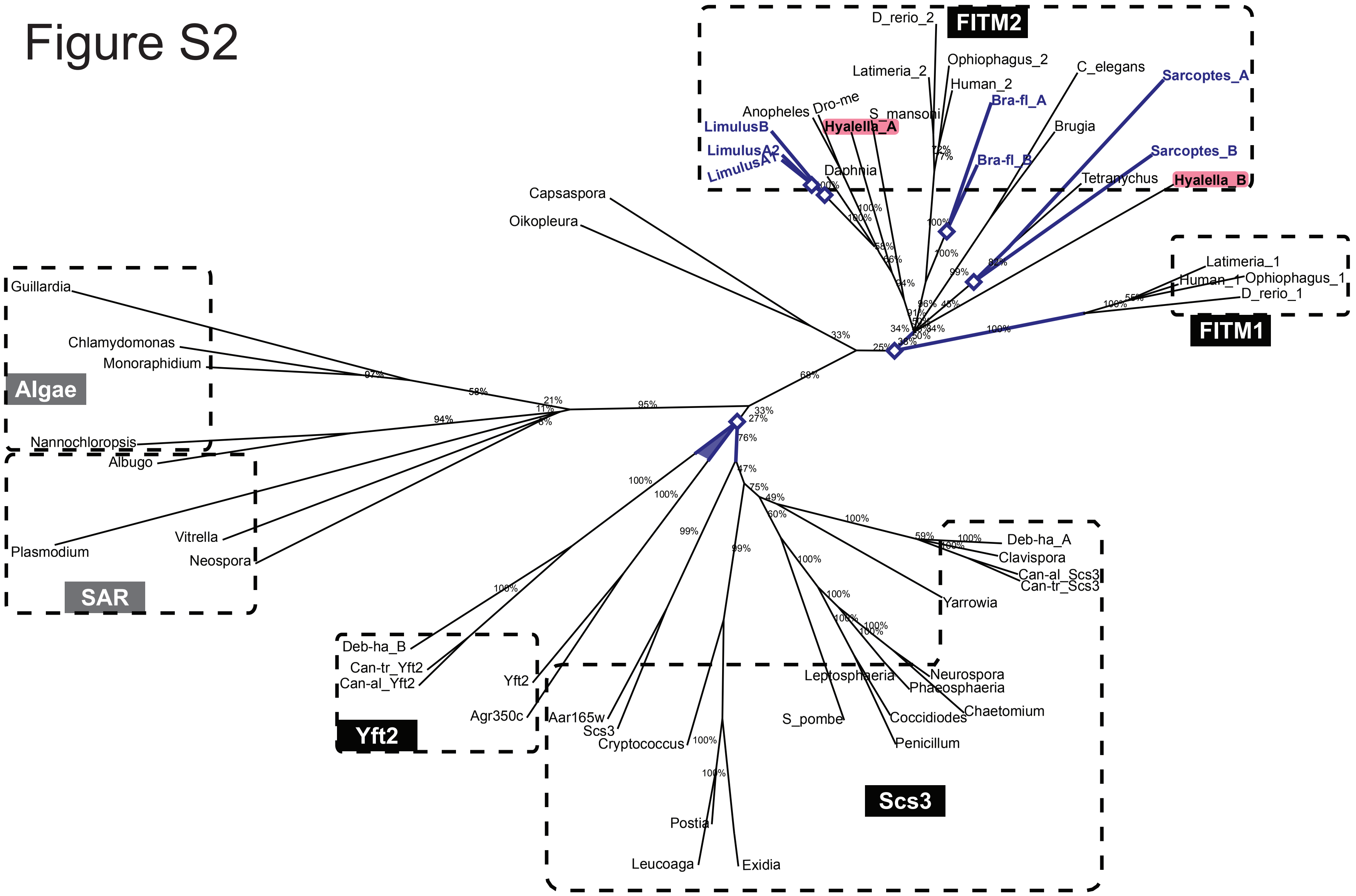
FITM2
FITM1
Algae
SAR
Yft2
Scs3
D_rerio_2
Ophiophagus_2
Latimeria_2
Human_2
C_elegans
Sarcoptes_A
Anopheles
Dro-me
S_mansoni
Bra-fl_A
LimulusB
Hyalella_A
Brugia
LimulusA2
LimulusA1
Bra-fl_B
Sarcoptes_B
Daphnia
Tetranychus
Hyalella_B
Capsaspora
Oikopleura
Latimeria_1
Human_1
Ophiophagus_1
D_rerio_1
Guillardia
Chlamydomonas
Monoraphidium
Nannochloropsis
Albugo
Plasmodium
Vitrella
Neospora
Deb-ha_A
Clavispora
Can-al_Scs3
Can-tr_Scs3
Yarrowia
Deb-ha_B
Can-tr_Yft2
Can-al_Yft2
Yft2
Agr350c
Aar165w
Scs3
Cryptococcus
Leptosphaeria
Neurospora
Phaeosphaeria
Chaetomium
S_pombe
Coccidiodes
Penicillum
Postia
Leucoaga
Exidia

Figure S3

A

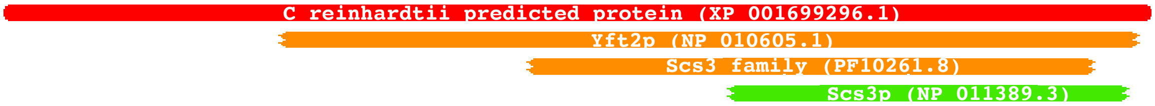

B

No	Hit	Prob	E-value	SS	Cols	Query HMM	Template HMM
1	**XP_001699296.1**	100.0	3E-118	36.7	311	1-311	1-311 (311)
2	**NP_010605.1 Yft2p**	88.3	3.6	7.4	211	76-307	3-267 (274)
3	**PF10261.8 (Scs3)**	88.1	2.9	6.4	136	143-295	68-223 (225)
4	**NP_011389.3 Scs3p**	74.0	91	10.5	108	197-304	223-377 (380)

where XP_001699296.1 is CHLREDRAFT_152504 [*Chlamydomonas reinhardtii*]

C

Hit number 2
>NP_010605.1 Yft2p [*Saccharomyces cerevisiae* S288c]
Probab=88.28 E-value=3.6 Score=40.86 Aligned_cols=211 Identities=18% Similarity=0.143
Sum_probs=77.7 Template_Neff=6.000

```
Q ss_pred               CeeEeCCHHHHHHHHHHHHHHHHHHHHHHHHHHhhhCCCCCCCCccc-------ccCcchhhccccceecc-chhh---HHH
Q CHLREDRAFT_152504  76 DKLVLPSRAALTALLSLPLLLWAGAVGAGCLHWESELPDGASKRD-------FEHPLIWFDRRVFRVWG-PLLP---LLP
Q Consensus          76 ~~l~~~s~~a~~a~l~~P~l~W~~~i~aG~~~~~~~~~p~G~tk~s----------~~~Pl~WfD~~vFr~~G-PlLP---L~p
                        .+..++++..+        ++.-..++.|++.+- -.|+. + .+        +-.+=..+. ++|.+-| ..--   +..
T Consensus           3 ~~~~~~~~~~l--------~iyp~~l~lG~l~s~~~~~~~~-~~~~~~~~~~~~Yfa~k~Ni~N~~~Fvk~g~W~WT~l~~~~
T NP_010605.1         3 RQLNYWSRKAY--------LIYPFQVFVGALLSI-VVSSE-T-LNHQKETCALLKSSNIFN-VIFAYKANQLWPFLFFSL
T ss_pred               cccccchHHH--------HHHHHHHHHHHHHHH-HcChh-H-hcccccceeccCCChhH-HHHHhccchHHHHHHHHH
Confidence              11122222221        223344556655331 11211 1 00       111111222 3555444 2222   112


Q ss_pred               HHHHHHHHH--hcCC-CCCCCC----------------ccCccchHHHHH----HHH-----HHHHHHHHHHHHHHH-HH
Q CHLREDRAFT_152504 145 FLIRVAALN--LRRR-PQPGGG----------------VLLRPAASLAVQ----AGL-----TYGIIIVVRLLLYGG-HV
Q Consensus         145 ll~Riaas~--l~~~~~~~~g~----------------v~lr~~a~l~~~~----ag~-----vY~~IaLvRL~vY~~-h~
                        .+..+....  .... +.+.+.                .+.+.-+.+..+    .+.     -+....+-|+.++.+ +|
T Consensus          71 ~~~~~~lt~~~~~~~~~~p~~~~~~~~~~~~~~~~~~~~~~~~~~~~~~~r~~~~~t~~w~~~Wffg~~l~dri~~~TGG~C
T NP_010605.1        71 AFLQIYFHYLARMDILPLPISSTETSSSYLTYTNHWPLLKNRIISIMITQYACKFVLKYLLLFLNFQFIDHVFIWTGGEC
T ss_pred               HHHHHHHHHHHhcCCCCCCCCCCccccccccccCCcchhHHHHHHHHHHHHHHHHHHHHHHHHHhHHHHHHHHHHhcCcc
Confidence              222221111  0000 000000                000000000011    011     111122336665553 33


Q ss_pred               HH--------------HhccchhHhcchHHHHHHHHHHHHHHHHHHHHHHHHHHHHhcCCCchHHHHHHHHHHHHHHHH
Q CHLREDRAFT_152504 196 AL--------------QRGAGVYLVSDHLLLAASVVACFQSELVLCFSDAHKSELLARHDPGAGAAQVAVVSGLVSSVFV
Q Consensus         196 AL--------------~r~~~~~~mSDHVFLgASV~a~~haElV~c~~D~~ks~L~~r~~p~~~a~~~~~v~~~av~s~~l
                        -.              .+|-+-+=.|.|+||=...+-.+--|+.... +..+     .+++....... .......+++
T Consensus         151 ~~~~~~~~s~~~C~~~gg~W~GGfDISGH~FlL~~~sL~L~~El~~~~-~~~~~-----~~~~~~~~~~~~~-~~~~~~~~~L
T NP_010605.1       151 SSGSKTTSAEKCRLENGKWDGGFDISGHFCFLVSISMILWMELHLFS-RFVQ-----AEDMFWVVNKW-VRACLAIVCAV
T ss_pred               cCCCCCCCHHHHhccCCEecCCCCChHHHHHHHHHHHHHHHHHHHh-hhhh-----hhhhhhchhHH-HHHHHHHHHHH
Confidence              11              2344456689998665554455555643322 1111     0111000000 00112344555


Q ss_pred               HHHHhccchheeecccCchhHHHHHHHHHHHhHHHHHHHHHHhhccC
Q CHLREDRAFT_152504 262 LVALYGDMYCTARWYHHPGENLASLGLGAVVFQLPVLVWLMRQARF  307 (311)
Q Consensus         262 mvlt~gDmy~TAR~fH~~~ET~aaavLG~l~fQ~Pvv~wl~~~~~~  307 (311)
                        +.+-..-.++|+-|||+..|=+...++|.+..-+  +-.+..++..
T Consensus         224 l~LW~~mLl~T~iYFHT~~EK~~G~~~g~~~~~~~--~Y~~~~~~~~  267 (274)
T NP_010605.1       224 LVIWICILWVTAIYYHTILEKVLGCLMGFICPVF--IYHILPKIGI  267 (274)
T ss_pred               HHHHHHHHHHHHHHHhCCHHHHHHHHHHHHHHHHH--HHhhcccch
Confidence              56666667899999999999999988764221  1111111111
```

Figure S4

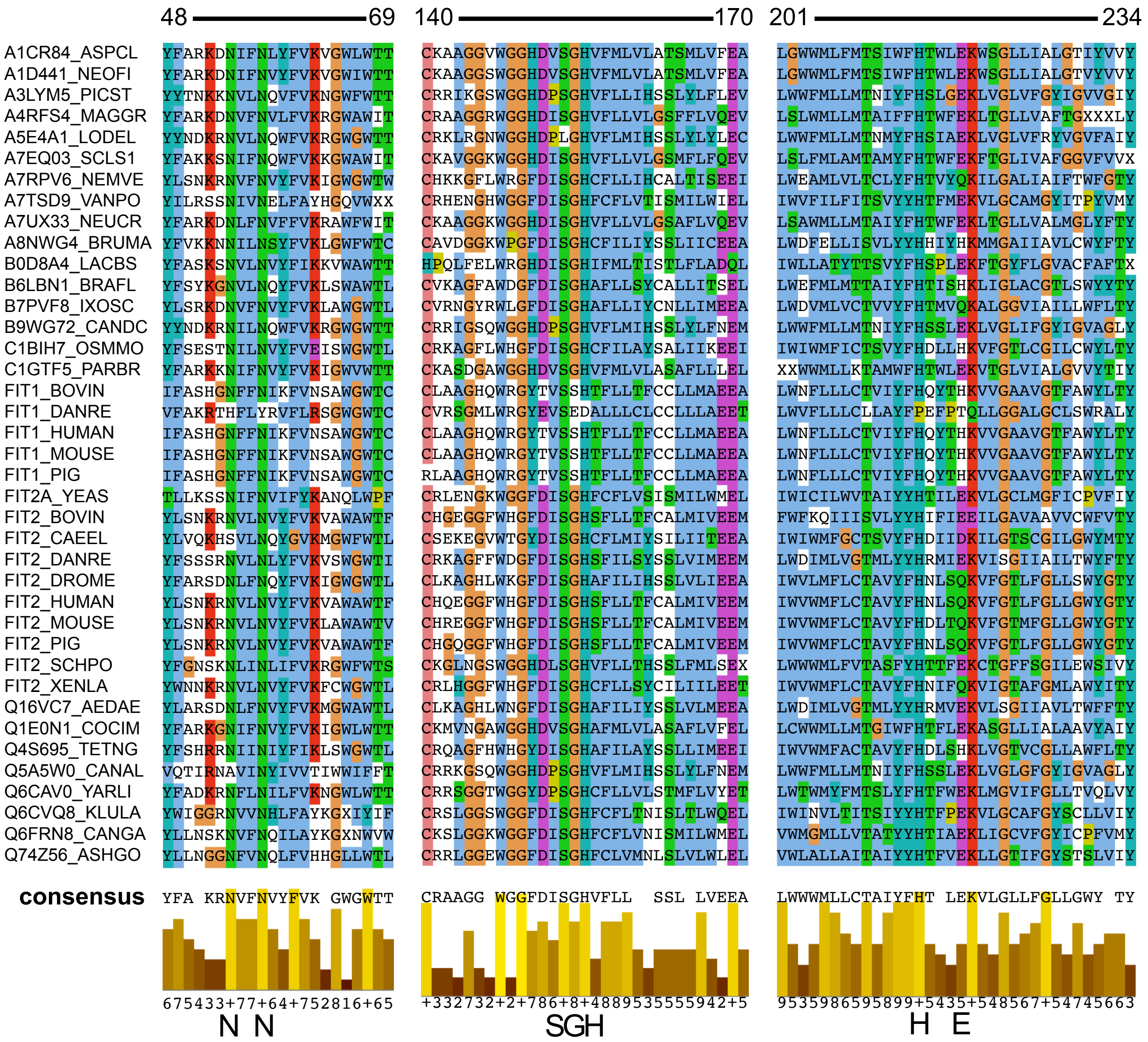
48
69
140
170
201
234
A1CR84_ASPCL
A1D441_NEOFI
A3LYM5_PICST
A4RFS4_MAGGR
A5E4A1_LODEL
A7EQ03_SCLS1
A7RPV6_NEMVE
A7TSD9_VANPO
A7UX33_NEUCR
A8NWG4_BRUMA
B0D8A4_LACBS
B6LBN1_BRAFL
B7PVF8_IXOSC
B9WG72_CANDC
C1BIH7_OSMMO
C1GTF5_PARBR
FIT1_BOVIN
FIT1_DANRE
FIT1_HUMAN
FIT1_MOUSE
FIT1_PIG
FIT2A_YEAS
FIT2_BOVIN
FIT2_CAEEL
FIT2_DANRE
FIT2_DROME
FIT2_HUMAN
FIT2_MOUSE
FIT2_PIG
FIT2_SCHPO
FIT2_XENLA
Q16VC7_AEDAE
Q1E0N1_COCIM
Q4S695_TETNG
Q5A5W0_CANAL
Q6CAV0_YARLI
Q6CVQ8_KLULA
Q6FRN8_CANGA
Q74Z56_ASHGO
consensus
YFA KRNVFNVYFVK GWGWTT
CRAAGG WGGFDISGHVFLL SSL LVEEA
LWWWMLLCTAIYFHT LEKVLGLLFGLLGWY TY
675433+77+64+752816+65
+332732+2+786+8+48895355555942+5
95359865959899+5435+548567+54745663
N N
SGH
H E

Figure S5

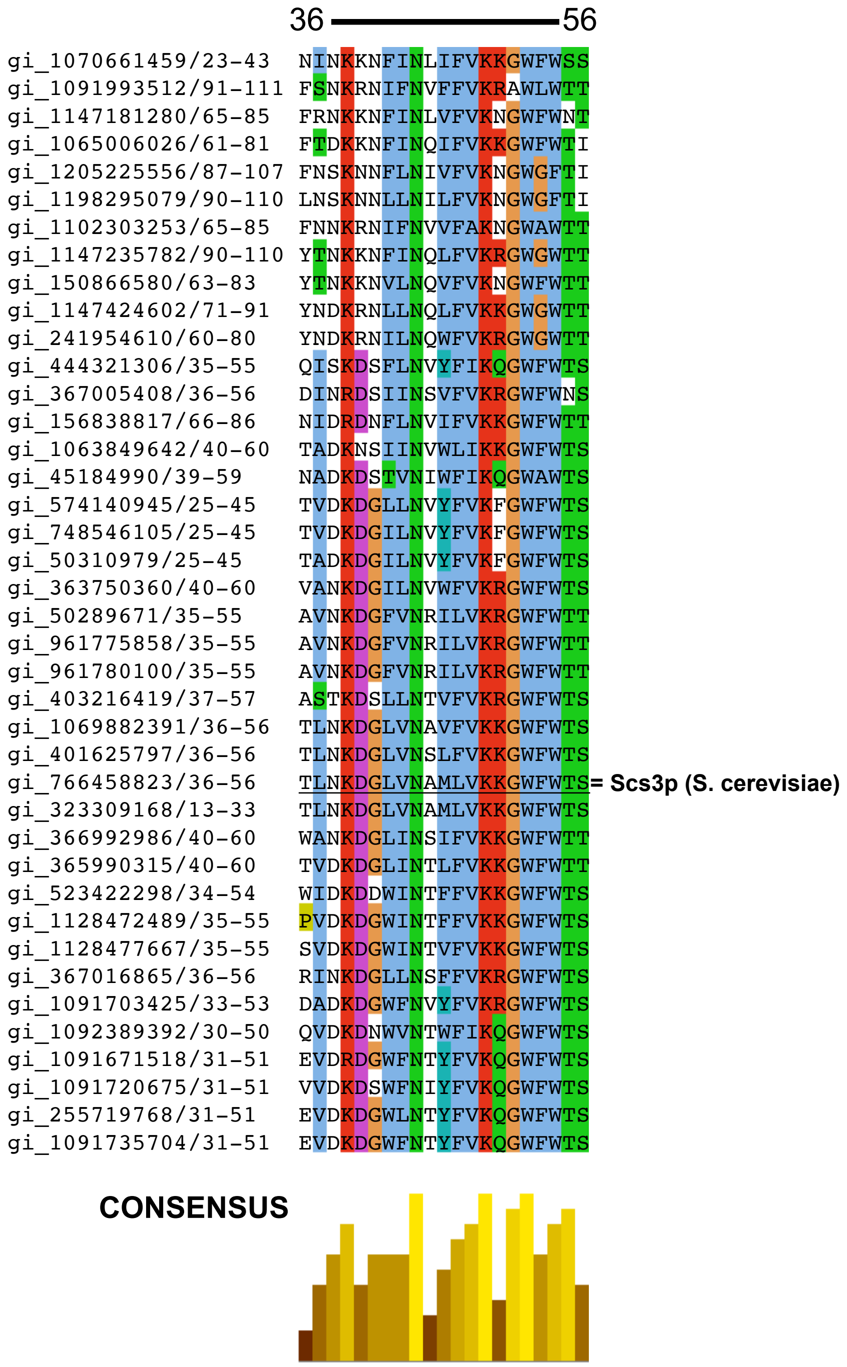

36
56
gi_1070661459/23-43 NINKKNFINLIFVKKGWFWSS
gi_1091993512/91-111 FSNKRNIFNVFFVKRAWLWTT
gi_1147181280/65-85 FRNKKNFINLVFVKNGWFWNT
gi_1065006026/61-81 FTDKKNFINQIFVKKGWFWTI
gi_1205225556/87-107 FNSKNNFLNIVFVKNGWGFTI
gi_1198295079/90-110 LNSKNNLLNILFVKNGWGFTI
gi_1102303253/65-85 FNNKRNIFNVVFAKNGWAWTT
gi_1147235782/90-110 YTNKKNFINQLFVKRGWGWTT
gi_150866580/63-83 YTNKKNVLNQVFVKNGWFWTT
gi_1147424602/71-91 YNDKRNLLNQLFVKKGWGWTT
gi_241954610/60-80 YNDKRNILNQWFVKRGWGWTT
gi_444321306/35-55 QISKDSFLNVYFIKQGWFWTS
gi_367005408/36-56 DINRDSIINSVFVKRGWFWNS
gi_156838817/66-86 NIDRDNFLNVIFVKKGWFWTT
gi_1063849642/40-60 TADKNSIINVWLIKKGWFWTS
gi_45184990/39-59 NADKDSTVNIWFIKQGWAWTS
gi_574140945/25-45 TVDKDGLLNVYFVKFGWFWTS
gi_748546105/25-45 TVDKDGILNVYFVKFGWFWTS
gi_50310979/25-45 TADKDGILNVYFVKFGWFWTS
gi_363750360/40-60 VANKDGILNVWFVKRGWFWTS
gi_50289671/35-55 AVNKDGFVNRILVKRGWFWTT
gi_961775858/35-55 AVNKDGFVNRILVKRGWFWTT
gi_961780100/35-55 AVNKDGFVNRILVKRGWFWTT
gi_403216419/37-57 ASTKDSLLNTVFVKRGWFWTS
gi_1069882391/36-56 TLNKDGLVNAVFVKKGWFWTS
gi_401625797/36-56 TLNKDGLVNSLFVKKGWFWTS
gi_766458823/36-56 TLNKDGLVNAMLVKKGWFWTS = Scs3p (S. cerevisiae)
gi_323309168/13-33 TLNKDGLVNAMLVKKGWFWTS
gi_366992986/40-60 WANKDGLINSIFVKKGWFWTT
gi_365990315/40-60 TVDKDGLINTLFVKKGWFWTT
gi_523422298/34-54 WIDKDDWINTFFVKKGWFWTS
gi_1128472489/35-55 PVDKDGWINTFFVKKGWFWTS
gi_1128477667/35-55 SVDKDGWINTVFVKKGWFWTS
gi_367016865/36-56 RINKDGLLNSFFVKRGWFWTS
gi_1091703425/33-53 DADKDGWFNVYFVKRGWFWTS
gi_1092389392/30-50 QVDKDNWVNTWFIKQGWFWTS
gi_1091671518/31-51 EVDRDGWFNTYFVKQGWFWTS
gi_1091720675/31-51 VVDKDSWFNIYFVKQGWFWTS
gi_255719768/31-51 EVDKDGWLNTYFVKQGWFWTS
gi_1091735704/31-51 EVDKDGWFNTYFVKQGWFWTS
CONSENSUS

Figure S6

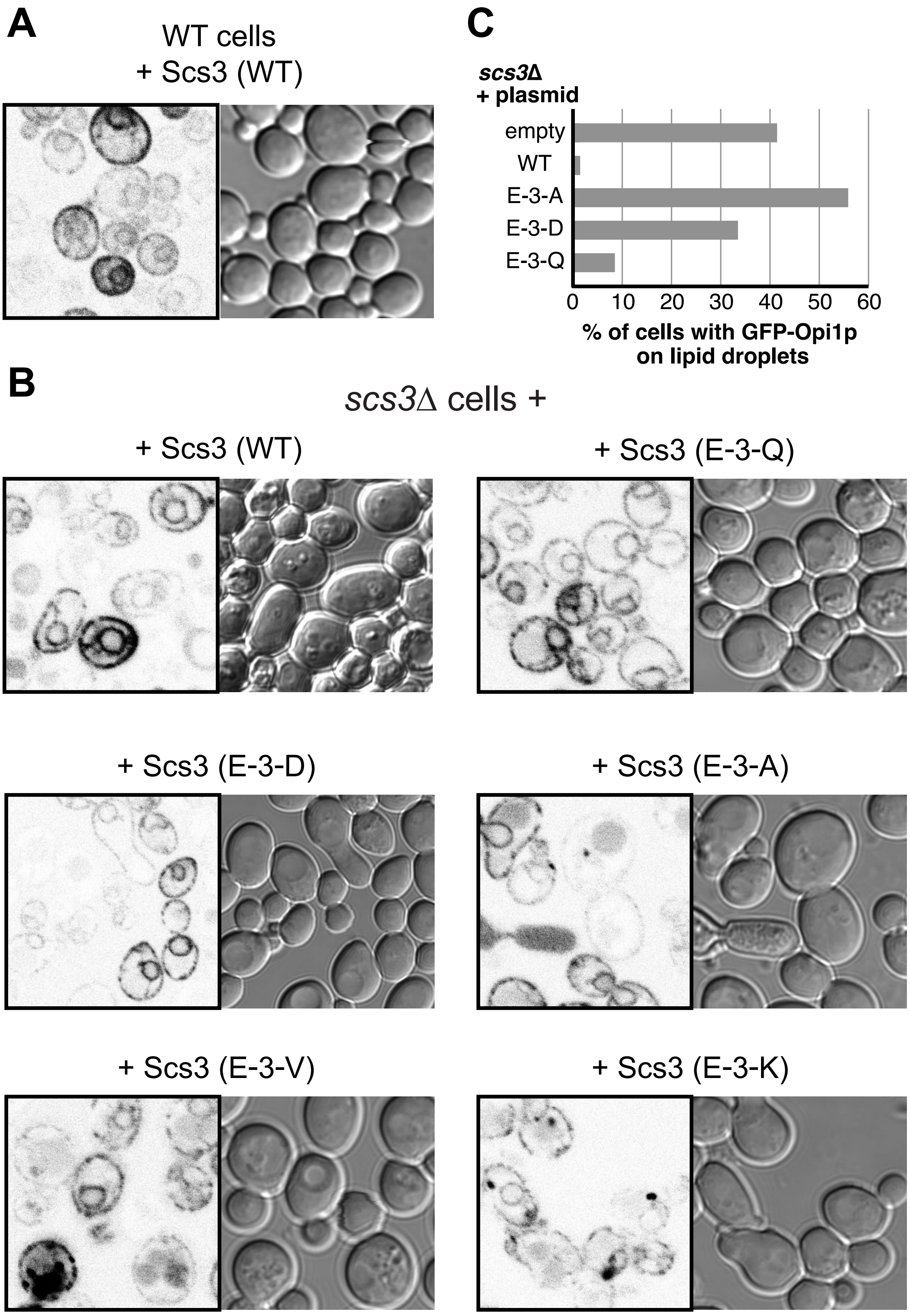
A
WT cells
+ Scs3 (WT)
C
scs3Δ
+ plasmid
empty
WT
E-3-A
E-3-D
E-3-Q
0
10
20
30
40
50
60
% of cells with GFP-Opi1p
on lipid droplets
B
scs3Δ cells +
+ Scs3 (WT)
+ Scs3 (E-3-Q)
+ Scs3 (E-3-D)
+ Scs3 (E-3-A)
+ Scs3 (E-3-V)
+ Scs3 (E-3-K)

Figure S7

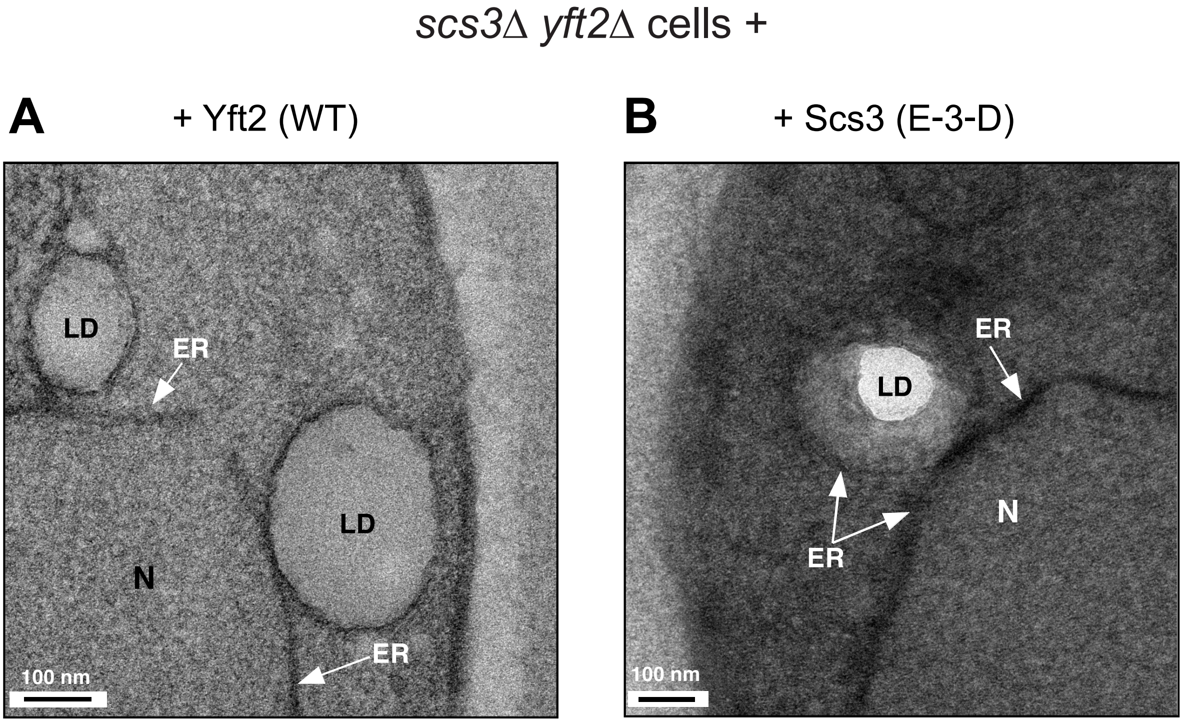

scs3Δ yft2Δ cells +
A
+ Yft2 (WT)
LD
ER
LD
N
ER
100 nm
B
+ Scs3 (E-3-D)
ER
LD
N
ER
100 nm

Table S1

1. hypothetical protein, conserved [Cryptosporidium muris RN66] 411 aa; EEA05309.1 GI:209555264

2. conserved hypothetical protein [Cryptosporidium parvum Iowa II] 384 aa; EAK90323.1 GI:46229505

3. inositol phospholipid synthesis protein scs3p [Cyclospora cayetanensis] 477 aa; OEH73834.1 GI:1069834430

4. inositol phospholipid synthesis protein Scs3p [Gregarina niphandrodes] 500 aa; EZG67081.1 GI:608673670

5. inositol phospholipid synthesis protein Scs3p [Gregarina niphandrodes] 500 aa; XP_011130335.1 GI:749157413

6. TPA: Inositol phospholipid synthesis Scs3p domain-containing protein [Neospora caninum Liverpool] 1588 aa; CEL69941.1 GI:820694201

7. hypothetical protein AM587_10007666 [Phytophthora nicotianae] 302 aa; KUF85555.1 GI:970644890

8. hypothetical protein L915_14512 [Phytophthora parasitica] 302 aa; ETK79652.1 GI:567954011

9. hypothetical protein L916_14419 [Phytophthora parasitica] 302 aa; ETL33070.1 GI:567982658

10. hypothetical protein L914_14361 [Phytophthora parasitica] 302 aa; ETM39498.1 GI:568042145

11. hypothetical protein F441_14758 [Phytophthora parasitica CJ01A1] 302 aa; ETP09387.1 GI:570944130

12. hypothetical protein PPTG_15377 [Phytophthora parasitica INRA-310] 302 aa; ETN04015.1 GI:568089165

13. hypothetical protein PPTG_15377 [Phytophthora parasitica INRA-310] 302 aa; XP_008910685.1 GI:675203601

14. hypothetical protein F442_14780 [Phytophthora parasitica P10297] 302 aa; ETP37412.1 GI:570975299

15. hypothetical protein F443_14945 [Phytophthora parasitica P1569] 302 aa; ETI39481.1 GI:566016570

16. hypothetical protein F444_14929 [Phytophthora parasitica P1976] 302 aa; ETO68223.1 GI:570320275

17. inositol phospholipid synthesis protein Scs3p [Toxoplasma gondii ARI] 1642 aa; KYF48237.1 GI:1005161369

18. inositol phospholipid synthesis protein Scs3p [Toxoplasma gondii FOU] 1641 aa; KFG56073.1 GI:672288519

19. inositol phospholipid synthesis protein Scs3p [Toxoplasma gondii GAB2-2007-GAL-DOM2] 1641 aa; KFG44054.1 GI:672271692

20. inositol phospholipid synthesis protein Scs3p [Toxoplasma gondii MAS] 1641 aa; KFH17838.1 GI:672580702

21. inositol phospholipid synthesis protein Scs3p [Toxoplasma gondii p89] 1641 aa; KFG50142.1 GI:672279353

22. inositol phospholipid synthesis protein Scs3p [Toxoplasma gondii RUB] 1641 aa; KFG66066.1 GI:672303231

23. inositol phospholipid synthesis protein Scs3p [Toxoplasma gondii TgCatPRC2] 1640 aa; KYK65535.1 GI:1008947497

24. inositol phospholipid synthesis protein Scs3p [Toxoplasma gondii VAND] 1641 aa; KFH09743.1 GI:672570415

25. inositol phospholipid synthesis protein Scs3p [Toxoplasma gondii VEG] 1642 aa; ESS35107.1 GI:557739206

FITM homologues in SAR protists identified in the protein database

Proteins annotated with Scs3p domains from organisms that are neither fungi nor metazoa (animals) were obtained at NCBI/protein by searching for "scs3p AND (alveolata OR stramenopiles)"

Table S2

A. PSI-BLAST with human FITM2, inclusion cut-off e-value 0.001, 3rd iteration

```
#   Protein identifier in NCBI, name of hit (with highlighting of "phosphatase")        e-value

1.  XP_002747619.2  PREDICTED: fat storage-inducing transmembrane pro...  1e-93
.
.
396 CDR39427.1  CYFA0S03e03180g1_1 [Cyberlindnera fabianii]ONH65653.1...  3e-05
397 SJM82338.1  related to FIT family protein SCS3 [Zygosaccharomyces...  4e-05
398 XP_011210325.1  PREDICTED: phospholipid phosphatase 5 isoform X2 ...  5e-05
399 XP_001543145.1  predicted protein [Histoplasma capsulatum NAm1]ED...  5e-05
400 XP_005190574.1  PREDICTED: phospholipid phosphatase 5 isoform X2 ...  1e-04
401 XP_019019411.1  hypothetical protein PICMEDRAFT_16102 [Pichia mem...  2e-04
402 ORX97661.1  acid phosphatase/Vanadium-dependent haloperoxidase [B...  6e-04


403 CQB89556.1  Inositol phospholipid synthesis protein Scs3p [Chlamy...  0.001
404 OJA14836.1  hypothetical protein AZE42_00788 [Rhizopogon vesiculo...  0.001
405 OAF67445.1  hypothetical protein A3Q56_04824 [Intoshia linei]         0.001
406 KZV63858.1  acid phosphatase/Vanadium-dependent haloperoxidase [P...  0.002
407 XP_011130335.1  inositol phospholipid synthesis protein Scs3p [Gr...  0.002
408 OEJ85630.1  hypothetical protein AWRI3579_g1773 [Hanseniaspora os...  0.002
409 XP_008883615.1  hypothetical protein HHA_313030 [Hammondia hammon...  0.003
410 XP_455512.1  hypothetical protein [Kluyveromyces lactis NRRL Y-11...  0.003
411 XP_001525569.1  hypothetical protein LELG_03497 [Lodderomyces elo...  0.003
412 XP_007881728.1  hypothetical protein PFL1_05999 [Anthracocystis f...  0.003
413 KNE70904.1  hypothetical protein AMAG_15000 [Allomyces macrogynus...  0.004
414 XP_017999866.1  Diacylglycerol pyrophosphate phosphatase 1 [Phial...  0.005
415 XP_010563273.1  PREDICTED: phosphatidate phosphatase PPAPDC1A [Ha...  0.005
```

B. Summary of phosphatase hits in PSI-BLAST with FITM2, iterations 3, 4 and 5

Iteration	Total significant hits (e-value <0.001)	new phosphatase hits / total new hits	most significant phosphatase (e-value)
3	402	3 / 402	5×10^{-5}
4	451	21 / 49	1×10^{-13}
5	813	176 / 362	4×10^{-21}

C. Residues found in 490 FITMs at +4 position following histidine in loop 5/6 (*i.e.* Hxxx?)

D	1.6%
E	78.2%
H	3.3%
Q	13.1%
S	3.3%
T	0.6%

D. Residues found in 1532 LPTs at +4 position following histidine in loop 5/6 (*i.e.* Hxxx?)

A	3.0%
D	86.5%
E	1.2%
G	0.1%
H	1.8%
Q	7.1%
S	0.3%

Supplementary File 1:

CLUSTAL Omega alignment of 57 FITM sequences

```
>Debaryomyces_DEHA2C02860/1-265
------------------------------------------------------------------------
------------------------------mklctwhiivvsyaiiyvasipwy--iflhqfkdkvsgnivi
aflnqnlivryg-yflftaayfseaitysrsel---------------------------------------
---------------------kisrsnllkilrhysiytl--ctvillg--wffgpsiyervnvltggycda
ntpkt-------------------------------------------------------------------
------------------------giagqfqciripgakwidgfdvSGHiyilstmclllw--esflenldl
rhf-sqqfegyt-------------nlrayf-s----------------------------kfdyeyspsip
tdeq-gyiiqteysnvirlvclv--ltvslliiwsmmyivtcv--ffHTLTEklvslivgiaislsitsln-
----------------------------------------------------------------

>CalbA_Yft2_CaO19.8750/7-297
--twgg--vwfs----vpfflvfcfdfs----vyvds-sk------------------------------st
ptt-----------------hnc--kmslryklspynialllyptvyigsiplt--lllkqynqyigqiffv
rfleqylvsnng-ylwfttlywlvvvftaylpn---------------------------------------
----------------------RNRdflifytkiylinti--wivvlle--wffgspiferisviagatcsi
r-----------------------------------------------------------------------
------------------------diyreyack-qsggvwtdpfdssshytylissslliw--hlllnhvtw
i-------sgyf-------------pkhvid-l----------------------------ensthyaap--
nsit-akdvkmdsnRTFRtiiev--iavfilimwfisytitsv--ffhtipekfvgllcglfvpivlkyik-
----------------------------------------------------------------

>Ctrop_Yft2_XP_002549187/1-252
------------------------------------------------------------------------
--------------------------mkltlkltpynfaylvypvvyvcsiplt--lllkryngtiahtfpv
nfiesyivsnhg-ymwftglywilaitlgylpt---------------------------------------
----------------------kdtviltrytkiylintl--wilille--wffgspiferisvfagatcsa
p-----------------------------------------------------------------------
------------------------gifreyeck-dnggewndpfdssshytflisssllvw--ylivthvsw
v-------tdyl-------------sshvpd-l----------------------------evgisyspv--
eetq-sk--vssayktvrtivei--vavvllgiwfisylttsi--ffhtipeklvglicglvvptlieyi--
----------------------------------------------------------------

>Neospora_CEL69941plus_PLC/1080-1495
yastlraafgnl----lpqffllsyesa----laaqe-at------------------------------et
asasq-gaaaagqgppsqgtesppphlspvlpvdrhavasdayap-lpsflpqssllfdfavfat-----pc
ypfsr-flhrrl-vswgglqfhillfggwihsl-----fapep-----------------------------
----------------------ltllhvpraff-lvlvsa------yrglvlyeglnwiekavwarnthcgr
peernpseastptt--------------------dketsg--------------------dgaqqepspdnr
gtatrsqateedgertrpdtsstedgassgdktnseg-gwwarkdlSDHvvlyvmaivfms--ievsaarss
hap--psfs---evpt---------pttgadasrrrarwftclppcl---------lp--------------
--r---kissflyilvf-----a--yygilflcclhmayytav--ffHTPEEiwagltvglcflvlpiilvl
evlekp------s------lqrigigsgek-kaaera--a---aaaaaasasaagaqnq-aggd

>Ashbya_Yft2_AGR350C/10-261
y--iseRRycyl----ypalllggevik----lilge-et--------------------------------
--i-------------------------------------------------eh--drktwylln-----gg
nfvnq-lfvhhg-vllwtllmglvlalq-yhv--rttefdplpldarqlgp----v----------------
RRh---pl-------------kmlgsmavqtvikvllv------vt----vlqalfwfmdhlfvwtggrctv
sd----------------------------------------------------------------------
------------------------t-kdsvacRRlgg-ewvggfdiSGHfclvmnlslvlw--leltelqry
mhsqeiqlr---------------------------------------------------------------
-------------kfawartt-----vlgalgvwlallaitai--yyHTFVEkllgtifgystslviyyvlp
RINRgl------hyl-------------------------------------------------

>Yeast_Yft2/7-274
y--w-srkayli----ypfqvfvgalls----ivvss-et--------------------------------
--l-------------------------------------------------nh--qketcallk-----ss
nifnv-ifayka-nqlwpflffslaflqiyfhylarmdilplpisstetss----sy--ltyt---------
nhw---pllk------n----riisimitqyackfvlk------yl----llflnfqfidhvfiwtggecss
gs----------------------------------------------------------------------
------------------------kttsaekcrleng-kwdggfdisghfcflvsismilw--melhlfsrf
vqaedmfwv---------------------------------------------------------------
-------------vnkwvracla--ivcavlviwicilwvtai--yyhtilekvlgclmgficpvfiyhilp
kigilh------nylyl-----------------------------------------------

>Auricularia_EJD35661/7-289
----pvqralal----vvattllgtays----vlast-wl--------------------------------
```

```
--d----------------tsnp-----------------------lianlphp--hhkasffas-----kr
nvfnt-lfvkra-wawtsllvaantlvnpRR-----------------------------------------
---------------------------apqriwrwalata--awlafas--wffgpslraraaalsgaecvv
qlpy-tptg---------------------------g--dvgahvltvpaeycvqrrpittaehahlfqqai
----gvas---------------------ipeswrgiprltrghdvSGHvfllslsiltlv--ddvatgapf
dal-----a--srt----------------------------------------------------------
-----------------qlaggv--ataalvalwywmllmtsv--yfHSPLEkisglvvgitaylftkipmp
tltapv------e----------gavsleK----KQQ---------------------------

>Exidia_KZV96244/5-296
----pvqralvl----vtavtllgtaws----tvakt-yl--------------------------------
--d----------------tsnp-----------------------lvanlphp--lhassyfan-----kr
nifnt-lfvkra-wawtsaaivlnlysnpRR-----------------------------------------
---------------------------apaRIWRfalata--cwaaftm--wffgpsvrarlaslsgaeciv
tlph-tts--------------------------------dgvhvltvpaeyciqrrtlstrehaevfqqal
lgntgaps---------------------ipddwrgvpkltrghdiSGHvfllslsiltlv--ddlftaapp
sga-----agfadt----------------------------------------------------------
-----------------qlataa--lssalcalwywmllmtsv--yfHAPFEkitglivgisaylvtkipfp
fegapp------visa----qpvpvvpiek----ktq---------------------------

>Crytptococcus_Scs3/60-421
----hqlilagv----vsslmlcgimys----lvhst-sl--------------------------------
--n----------------tsei-----------------------hhnhipe-----rvayfar-----ks
nilnv-ifvkra-wawtsaiyflhlvtspRNRssmf------------------------------------
-------sfs------t-----spnggRIRRllvwvaata--fwaifar--wffgaglgdriialtggncal
plppsispilarht------------fsklftagpqgssgeekiyialpykfct-gvpltpgalpelfallp
gggkgtai-----------ppgggptasheslaplprprwhkgfdiSGHsflltlcimvlg--relaptwq-
-aw-----aggktagl--------l-------ngRGRgw---------------------------------
-------------kgkihglial--aatgligiwmwmvlmtav--yfHNPPEklaglllgllssglinlfip
tfspsp------fdpitttrtstppdsgpdsregeRR--p---fshs------lgd-------g

>Postia_EED81670/4-290
----vriavlvv----itsivlfgtlys----vvnst-yl--------------------------------
--d----------------tsnp-----------------------lltylphp--lhsthyfas-----kg
nilnv-yfikrv-wgwtsaaflahyltsppa-----------------------------------------
-------------------------artkervlqflaata--vwltftg--wffgpavldrlvaftggecvl
glpsgdvvs--------------------------------------vpadycytkstlsvathpalfpaa-
--l---ll---------------------pedgwrgrprlrrghdvsghvflltmstllla--dqlrasfg-
-ra-----vgrws----------------------aphw---------------------------------
-------------wam------a--fgvgvvvlellavyttsv--yfhtpfekltgyllgvagfaitqlpv-
-fqpap------eavvvsnr-evpreagekaeiakrq---------------------------

>Leucoagaricus_AN958_11437/4-261
----pRRaafli----ltsvivfgtays----vlydt-yl--------------------------------
--d----------------tsdp-----------------------sishlphp--lsnshyfar-----ks
nflnv-yfikka-wgwtsalffllwttsppe-----------------------------------------
-------------------------nRTARRalkwaiatc--vwimftm--wffgpslldriivasggacvv
rlpsgelat--------------------------------------lptdacfagstvgpashphffssa-
--g---el---------------------stsnwtalprlrkghdvSGHvflltmstlfla--dqllignl-
-sl-----igvw------------------------------------------------------------
----------------------------------ilasyttsv--yfHSPWEkvtgyllglagftltqllv-
-tRGRs------saqvsiem-qhssk--------------------------------------

>Yeast_Scs3/4-380
k--wfnaihllv----cpltvlvgylmn----aygy------------------------------------
--------------------------------------------------------gaalqatln-----kd
glvna-mlvkkg-wfwtslvgwwci--iRYRav---------------------------------------
------pgat------g-----RDRRhivqsfkryailtv--wwyvftqgiwfgvgpimdlvfvytgghchy
dvfddaghvnedfqgsvtRTNRalalihnvlt--lhghhqehrq--qqlwdrsigsi---qqalqatqpktp
knvtasaaaaintfihdqmhrwqgplttsaqcRRfgg-hwagghdpSGHvflatlmcmfll--gelRVFGRR
a--lahlya----qkwqlvr----lvtrlfd-tgplwtwRRcgggsm---------tcgaRLWRaiveppvt
ca----aallRLTRciacdhpvi--illtllvtwlwqllltavasrfHTVREhmsgllaayivtglvyarda
aalrpv----------------------------------------------------------

>Ashbya_Scs3_AAR165W/9-362
S--RFLWVQLSL----CPVIIVVGTVMR----IFSE------------------------------------
--------------------------------------------------------PNSW--NAD-----KD
STVNI-WFIKQG-WAWTSAVCWWCV--VRYKGL---------------------------------------
------GGRS-----------------LRQTALRYVALTA--WWYAFTQAIWFGIAPIMDQVFTSTGGHCSF
DVFDTGMGLNSGFHDSEGRRTRSLQKLLQWFT--AHGAQGDELRERTMYWIRCML-----TGGQCE------
--NTGLDPTELNSYIQDSVT--ASAIRSSHACSRLGG-HWMGGHDPSGHVFLITLMCMFML--GELQVFGRR
A--IGKLSA----DCQQLQGAPGKIVARILQ-ASPIRELINSEAPQ-------------hILRRLFVQLPLE
SLAILVSAVVFGFRFIVLENPIL--LLVGLILTWTWSLLVTIL--SFhSFAeHLTGLLFAYLLVLALYWYI-
```

>Calvispora_CLUG_02545/30-293
----sefifafs----filnfivgkllh----vfs-------------------------------------
--qk--eevynyynd-----kg
nifnq-wfvkkg-wawttgaialfytiev---
-------sks------g-----frlkvlrgavlrwlvatc--wwylftq--wcfglpimdkvfvltggkcas
vsaeklarlteslh--
--------l-----ftlvdgvyessaisssqcrrlkg-swegghdpsghvfllvhsslymf--heikpfwpg
wkslyrsiirftrntnkdn---
--------vmaksralvvsnpsi--lvlgllglwwfmllmtnm--yfhslaeklvglvfgyiavaaiywl-p
rwm---

>Debaryomyces_DEHA2C09240/81-359
----geliflvs----fianfflgklih----lta-------------------------------------
--eh--eevynyynn-----kr
nvfnq-vfvkrg-wgwttliivvfysflmygnshaRIR--tkq-----------------------------
----------------------qrisvlkkaifnyvvatl--wwvlftq--wcfglplmdkvfvwtggkctg
iaqeklalhipsyg--
--------tssifteiesdlsyeskaitsymcrklkg-swegghdpSGHvfllihsslylf--lealpfwis
watlkhhlcqfvkswrstsgsk--
---------pkllfglfaqdpht--iiiplislwwfmlfmtni--yfHSIGEklvglvfgyvglavvyyv-p
rwl---

>CalbB_Scs3_68477565/30-333
----yeflfais----flvnfilgrfih----fsa-------------------------------------
--pd--eevynyynn-----kr
nilnq-wfvkkg-wgwttlviilfysniiykqynskat--tnttttnnnn----------------------
------nnkq------g-----vivqtirnavinyivvti--wwifftq--wcfglpimdkifvltggkcsi
dtnsmahfnpnhhh--
--------vhanf-vqkleniwestgitsyncrrikgsqwigghdpsghvflmihsslylf--neminywpg
wtyikhnlsqiltsgndngndngngngngt--
-----rslsigdkllllwntpql--iiggliglwwfmllmtni--yfhssleklvglgfgyigvaglywi-p
rwll--

>Ctrop_Scs3_EER30745/28-290
----gefifvls----filnfligriih----fts-------------------------------------
--pe--eeiynyysn-----kk
nifnk-lfvkqg-wgwttlvilifysnliyktsnysi-----------------------------------
-----------------------rqkliykgilnyllstl--wwilftq--wcfglpimdkifiwtggkcki
egieikn----inh--
--------lhnsf-iqtleniwestgitsytcrkfkg-nwigghdpsghvflmihsslyly--letidyfpg
fsniknnlkRLVR---
------vnsfkekliilwntpsi--fislliglwwfmllitnm--yfhsileklvglifgylgiaviyyl-p
RWR---

>Yarrowia_YALI0C24145/120-392
-----------s----lldlsliglvfs----vlilg-al--------------------------------
--v---gk--ivdhtyfad-----kr
nflni-lfvkng-wlwttiafgyivyetfsgsiglgvsfgtntstgtrtgt----thdgvsetnsdskan--
aal---kllv------g-----isaptpigqlsryiiHSL--WWLLFtq--wflgipimdrffvatggkcey
qkenasp---
---------------------itgktissascrrsgg-twiggydpsghcfllvlstmflv--yetiphikr
r---
----------------pyklsak--ialgtaalwtwmyfmtsl--yfhtfweklmgvifglltvqlvyvvip
ylt-rp------k----------psvgsnn----------------------------------

>S_pombe_SPBC543.08/31-250
-----------------attlllgsiys----iyvdk-----------------------------------
--wsitsyfgn-----sk
nlinl-ifvkrg-wfwtslvyfyhawdq--
----------------------krnkidfkfisryivatl--wwmfvtq--wfigpglidrtfalsggsckn
fdgds---
---------------------svfipltastckglng-swsgghdlSGHvflllthsslfml--senfsfiln
----
--------------ngikatstk--vlfgllglwwwmlfvtas--fyHttfEkctgffsgilewsivyvf-s
srmpav------a----------dllgssdy---------------------------------

>Neurospora_EDO65004/76-407
-----------------ptlllfgtlfs----llnps-ar--------------------------------
--ssp-ydf-------------v-----------------------sqshvqsg--daipsyfar-----kd
nlfnv-ffvkra-wfwitvsffgflfshpgyn-----snnnnnmtgtrggd----n----------------
----------------g-----sgtgrkikavvrwglvtl--wwvfvtq--wffgpaivdrgfrvtggkcqe

aqggrvnaqvkadam---
--------pgp--vgsnvevaglkefvtaaackaagg-kwqgghdiSGHvfllvlgsaflv--qevgwvvar
h-----ywRRSVRDERtvvmgdgavksagtd-laadkrw-------------------------ddyspemd
eaREVRdvvtggglwdalghggk--vvfvvvglsawmllmtai--yfHTWFEkltgllvalmglyftyiv-p
rfvpal------r----------givglpgv---------------------------------

>Chaetomium_CTHT_0021850_plusAdenylylT/25-325
-----------------palllfgalfs----fvspe-tr--------------------------------
--asp-ydp-------------v-----------------------rqshypp---ekapsyfar-----kd
nilnv-ffvkrg-wawitiaffvfwathpavt-----rk---------------------------------
-----------------------dwgsrikavmrwasvtt--wwifvtq--wffgpglvdrgfmltggrcer
eigelia---
------------------------evfsasackasgg-kwkgghdisghvfllvlgsyfli--qevgwvvvr
s-------tkavedercvlmddgaiksaave-appdkre---------------------------------
------dvqvltnvfealafggk--faavviglsvwmllmtai--yfhtwfekltglltalaesqapvss-r
sssqgp------sgsksprtfrdnmtalpdsmssvkv--p----an-------lp---------

>Phaeosphaeria_Q0U0J9/45-321
-----------------pltlllgsifs----tlspt-sr--------------------------------
--sap-ysa-------------d-----------------------sqshlp----efapsyfaq-----kk
nvfnv-yfvkvg-wfwttlafgvfvgfhpgfg-----a----------------------------------
----------------------gisrrrvaaiiryvvitg--wwvavtq--wffgpplidtmfrftggqcer
lrdp--
--------------aermdmsdtrefitaatckavgg-twkgghdisghvfllilgssllw--leflpaltr
--------veglrdgrlitladgktasvave-kervkve---------------------------------
--------------gdatargvk--falgvaglmwwmllmtaa--yfhtwfekftglavaftglwvgvlyaq
ggwrrs------r----------pvlgmpgv---------------------------------

>Leptosphaeria_LEMA_P018470/182-457
-----------------PATLLLGSIFS----IVSPA-AR--------------------------------
--AAP-YSA-------------D-----------------------SQSHLP----EFAPSYFAQ-----KK
NVFNV-YFVKVG-WFWTTVAFAMFVTLHPSFG-----R----------------------------------
----------------------GFSKRRVQAVLRYAAVTT--WWTFLTQ--WFFGPPLIDRGFRFTGGMCEL
IRDP--
--------------DARADMSDAKEFITAATCKAVGG-TWKGGHDISGHVFLLILGSALLW--LEFLPALTR
--------MEGLRDGRLITLADGKVASVIVE-KERVKEE---------------------------------
--------------GDATTKGVK--FALGVAGLMWWMLLMTAA--YFHTWFEKFTGLLVAFAGLWTVYFL-P
RGVPQI------R----------TWLGMPGV---------------------------------

>Coccidioides_Q1E0N1/31-309
-----------------pitlllgsfys----lispt-ar--------------------------------
--p----------ltpnlssat------------------------saphhp----pspvnyfar-----kg
nifnv-yfvkig-wlwttvaflsilstqpafv-----srridp-----------------------------
----------------n-----krlrriyqalfryavvtl--awvlttq--wcfgpaiidrsftatggrcer
ihas--
--------------gmkeai--sdsimtamackmvng-awngghdvsghafmlvlasaflv--fellgstvs
--------vdekndag-datddgaraeipv----nesgq---------------------------------
--------------spaaklsrn--fvwavaglcwwmllmtgi--wfhtflekasgllialaavyaiyll-p
rsspsw------r----------nvigipgr---------------------------------

>Penicillium_XP_002144088_plusGAL4/35-336
-----------------pltlligsiys----vispt-ar--------------------------------
--ssq-nfgsagpltptiatdvn-----------------------vptehq----pnpvnyfar-----kn
nifnl-yfvkig-wvwitaaflallltrpyyt-------rvpg-----------------------------
----------------n-----lrgkrsvraflrytivts--awflttq--wffgpaiidrmfvitggkcer
lpav--
--------------ipddtwegvkviltaaackasgg-lwrgghdvSGHvfmlvlgsasla--lealgtsps
--------sgsntses-gqvhdkd-gsdge----ntaag---------------------------------
--------------dpiskystk--fvwiviglswwmlfmtai--wfHtllEklsgllisvsvvyityfl-p
rnlpsw------a----------diigipgyepaslq--y---fas-------lgp-------s

>Plasmodium_XP_001347817_plusDNAtopo/453-691
----ltkipfyff-------------md--------d-yy--------------------------------
---dt----pcyvyskl-----is
nllrn-yykgif-lyfiifigcvhvv--------------------------------------fspf----
-------------------------pitlvhfrrffiyfiiimyrlfil--yfi-----psiiqyv------
----
----------------------iykf------hnfke-eyihifdySDHvil--fctllfiisleikaie-y
-----tikhq---ess--
-sdhfhf--kynrnfcffflkfvlyyyyilisfflytsyftsk--ffHTTNEifvayffstfsiffffyff-
-lykny----fsfys--igi--------tsymk-knnpapsnvfhtp-scl-si----------

>Sarcoptes_2_KPM11571/7-303

```
----pkrlilylf---si--ilfgsikd----yfldd-sq------------------------------ia
----------------------------------------------hsnhsvid--svlsyikse-----kk
sflnl-yfvkwg-wlwtwialipfliftrivfvlnqik---kk----qqqk----nysti-nlsassqsnat
t------nln------eieswyhyrseiigpiirqilttl--vwkfsvq---lf-----fdisvwt-ghcqt
knn---------------------------------------------------------------------
----------------------iflhktvqecrkegn-twiygldiSGHiflmtfsslvii--eelrflldk
fsefnef--s----------skqrllgvstanrs--------------------------------------
--hkyqf--kldsfvhwitlilt-siqilvifiwdymmiqtnl--fyHNLIQkigafiwavffwfiiyrip-
--lsyv--------l--pv---------------------------------------------

>Hyalella_XP_018013278/31-326
----ymlvpwyaragatilvvavlsflq--------g-ad--------------------------------
---------------------------------------------------ei----khvdalts-----kd
silnq-vfvkmg-wawtllaflvl---tlsllplspqp----------------------------------
------------------------lnasikmalralllti--ifytwca--vif-----psiehyt-gmcls
nnv---------------------------------------------------------------------
----------------------vvsrhdkkkclksgh-kfd-sfdisghaflmvycvltim--qeaqtmRRy
malgqgigkl-nenpd---agnanvqgnstmtkenaqnllasdqpkssedinkvasspvqenlealpdse--
-vikfqa--tymrlwpivsiayv--gvcvlcvmwdivlvitti--yyhtlsekllgisiaassfaflykyl-
--fikl------nll-------------------------------------------------

>Oikopleura_CBY36204/16-243
------vsswil--------vlllsvts--------i-lv--------------------------------
---------------------------------------------------ii----pss-ffdn-----pd
nilnt-lfvkwa-wgwtmysllpavvlvswaigddfr-----------------------------------
------------------------qnslraalkivffgtt--gwflgtq---lt-----fkigdyt-gtcvd
snfs--e-----------------------------------------------------------------
----------------------v-sfgtkrlcrsngh-twd-yfdiSGHtfllswsvyiin--aellep---
--areffkkt--------------------------------------------------------------
---------hsfllevitllaiv--wngllvilwilmlistql--yfHTIGEkilakfladv-ffvlyrlag
elvnr-------llp---lt--KAKNY-------------------------------------

>Oikopleura_CBY1889/16-264
------asswil--------vlllsvts--------i-lv--------------------------------
---------------------------------------------------ii----pss-ffdn-----pd
nilnt-lfvkwa-wgwtmysllpavvvvswaigddfr-----------------------------------
------------------------qnslraalkivlfgst--gwflgtq---lt-----fkigdyt-gtcvd
snls--e-----------------------------------------------------------------
----------------------vssfgtkrlcrsngh-twd-yfdisghtfllswsvyiii--aellep---
--areffkkt--------------------------------------------------------------
---------hsfllevitllaiv--wngllvilwilmlvstql--yfhtigekilakfladv-ffvlyrlag
elvnr-------llp---lt--kgrsg-ecfis-etk--p-itsnkt-kqk-h-----------

>C_elegans_FITM/46-283
----v--alfyl----af--vtvlsfie--------s-ri--------------------------------
---------------------------------------------------el----dstyylvq-----kh
svlnq-ygvkmg-wfwtlvivgpfiwfsskahnrrdr-----------------------------------
-------------------------dqpivdvcrlgvgta--cwyfsvq---ff-----hkvlalt-smcdk
gr----------------------------------------------------------------------
-------------------------tltraqcsekeg-vwtpgydisghcflmiysiliit--eeaiayrhy
qqvtdavhqm-dgd----------------------------------------------------------
-----re--ehdrltrciqyffv--amlflhafwfkqiiisvl--yyhifieeilgavaavvcwfvtyrml-
--ypag------fl----as--pirrtvgrk---------------------------------

>Brugia_malayi_FITM/43-273
----k--aifhl----la--vfvlslfa--------a-fv--------------------------------
---------------------------------------------------pl----pnhyyfvk-----kn
nilns-yfvklg-wfwtcvvvcpfiwyistavgqs-m-----------------------------------
-------------------------sgiiknlsrmviatt--iwyycth---sf-----vvfeqmt-ghchg
sk----------------------------------------------------------------------
-------------------------lssrsscavdgg-kwipgfdisghcfiliyssliic--eealafrni
tiihrtrk-m--------------------------------------------------------------
---------psvkngnliriffl--smcvlhllwdfellisvl--yyhhiyhkmmgaiiavlcwyftyhiw-
--yrkv------gip---pl--plq---prkli-------------------------------

>S_mansoni_Smp_018210/58-333
----kag--vyl----cl--aagvslvf--------d-fv--------------------------------
---------------------------------------------------ra----ppt-yfsn-----kq
npfnr-yfvklg-wawtcgclaafiivssfvytagni-----------------------------------
-------------------------klmrghilrlvvgsg--cwytvtg---li-----nlihdws-ghchp
vsvtlpn-----------------------------------------------------------------
----------------------glrpnqrischragg-vwl-gfdiSGHcfllimsnlwii--eelscmqhw
nklseilqln----------------------ks--------------nepn---qstntsgirhvsqqe--
```

```
-lnimrs--ayrrlttmirlifs--ftaclsmlwdimflstvi--yfHTMPSkllgtalgvacwflfyrvi-
--frscptgywggfa--pgl--pgdgp-iKFVL-S-----------------------------

>Hyalella_XP_018026323/63-352
----kit--lys----la--lffgslvf--------d-fv--------------------------------
---------------------------------------------------pm----prt-ymss-----ke
sifnv-yfmkig-wawlifivgsfvyltsatygcgkr-----------------------------------
-------------------------niiqqhmirliigtg--mwglmtk--wlf-----vtleqat-gtclg
ka----------------------------------------------------------------------
------------------------aiddkwlcvtsgf-qwh-sfdvsghaflliymnlvtv--eeknsrigw
gsrssgaeng-agsss---aesgswssssgqnkt--------------esdh----ssaetplrsltnne--
-fstfke--hyevftpyirvlfi--cmtllslwcdvllactvv--yfhtmpqkvvggacamaawyityrvm-
--fknq------w----pgl--pgdgglvryqg-tke--kpkev--------sq----------

>Anopheles_gambiae_FITM/76-364
----kvp--lyl----gs--lfivslig--------d-fl--------------------------------
---------------------------------------------------py----pkt-ylar-----td
nlfnv-yfvklg-wawtllfafpylamtsiticcgdn-----------------------------------
------------------------qrlirnhlprlgiatv--fwfvwtk---lf-----nviessy-grcsv
r-----------------------------------------------------------------------
------------------------gfdaktpclkagh-lwn-gfdisghafiliysslvlm--eearpiigw
esikdllrne-------------------ehnrs--------------nndn----sqtsnplknlkded--
-lkalky--fynrftptirlffi--gmtmlqllwdlmlvgtml--yhhrmvekvlsgiiavvtwfvtyraw-
--yplp------tvl--pdp--vgkgl-fnyqs-isk--peiglrrr-asl-lqpgsta-aaga

>D_rerio_FITM1/439-725
----kva--lyl----gs--lfvisvig--------d-fv--------------------------------
---------------------------------------------------pf----pkt-yfar-----sd
nlfnq-yfvkig-wgwtllfvvpflvlsaytitcgdh-----------------------------------
------------------------krmlrhhfpriviatf--fwffwtk---lf-----nvvensy-grctt
k-----------------------------------------------------------------------
------------------------gyatkssclkagh-lwk-gfdisghafilihsslvli--eearpiirw
etikehirne-------------------rhnrs--------------taen----s-gtnplrtlneeq--
-mrslqf--lykrltpiirtlfi--gmaalqllwdimlvgtml--yyhrmiekvisgiiailtwyftyrfw-
--yptp------gll--pea--pgngs-fsyqr-ei---ptfpfkrp-shl-stgaatt-ssgs

>Daphnia_EFX73912/39-314
----kvg--iyi----ml--vffgsilg--------d-vl--------------------------------
---------------------------------------------------pi----pns-yfsr-----kd
nmfnv-yfvkls-wgwtitcvgsfvyltssvyscgdq-----------------------------------
-------------------------tkirkhmlrllfatf--mwlfwtn---lf-----vnieesy-gycsk
aii---------------------------------------------------------------------
-------------------------rsnkkqclskgl-mwn-sfslSGHtfiliyctliim--eeakalisw
eaikdhlrne-------------------dhnrs--------------neen----v-satpldslsaeq--
-llyvre--kyekftpyirisfv--lmtmlaviwdvmlmatii--yfHSTPEkfvagviavllwffsyrfl-
--fnrk------llg--vpl--pgegt-frymn-pfq--plqtmhlR-R---------------

>Limulus_XP_013777312/53-316
----kvl--iyi----gt--vflisgic--------d-if--------------------------------
---------------------------------------------------psa--isks-yfsr-----kd
nfinq-yfvkrg-wgwtllivglfifltsrtycrgdk-----------------------------------
-------------------------kliskhmsrlliatv--mwfsctf---lf-----eyiewlt-gkcek
r-----------------------------------------------------------------------
------------------------dietkdlckksgh-ewq-gfdiSGHtflliycgliim--eegrcitvw
egigkqivqn----------------------yv--------------fean----i-rnstlRRlsene--
-iselrk--sykkltpfvqltfv--lmtilllmwdvmlmativ--yfHSIAQksvggiiatgawlvtyckl-
--frmk------h-s--pgl--pgegl-lRFQR-nsa--w------------------------

>Limulus_XP_013776821/61-327
----kig--iyv----si--lffvslvc--------d-fv--------------------------------
---------------------------------------------------pf----pes-yfsr-----kd
nllne-ylvklg-wgwtltivgtfvyltswtyccsdk-----------------------------------
-------------------------vlvrqhlsrmlvgtv--vwffctt---lf-----qymeewf-ghcyp
peh---------------------------------------------------------------------
----------------------gknyktkglcrkhgl-kwv-gfdiSGHaflliyccliim--eegkcirgw
erigelivrn----------------------ne--------------fed--------esplkklseeq--
-myklkt--syeqftpyvrvtfv--fmtvllliwdvmlmttil--yyHNMVQklaggliaigiwfityriw-
--yrmk------y-s--pgl--pgegl-fkysh-lks--prdis--------------------

>Limulus_XP_013782375/62-323
----kic--iyt----gv--vffaslic--------d-fl--------------------------------
---------------------------------------------------pf----prs-yfsr-----qd
nfmnq-yfiklg-wgwtllvvavfvyftsriyccndl-----------------------------------
```

```
-------------------------vlirkhmsrllvgtl--vwfcftt---lf-----eyieiwt-aecqv
rgq---------------------------------------------------------------------
----------------------gkkyktkdlckengh-kwm-gfdiSGDtflliyccliim--eegkcikgw
erigeliirn----------------------de--------------fed--------esplktlseeq--
-mselkk--syeqytqyvrltfv--lmtvllllwdvmlmattl--yfHNMVQklvgnvfavgvwfatyrlw-
--yqvk------l-s--pgl--pgvgl-fnyld-lqn---------------------------

>Capsaspora_KJE96303/36-294
----gihlpwvvvlqaiv--vvfgsvla-----------n--------------------------------
---------------------------------------------------ld----aeh-yfad-----kt
nilni-mfvkrg-wawtvgllvvfklavvldgrq-s------------------------------------
-----------------------w-rhfsnvcarlaiata--gwwcvtt---gl-----dtvrhwt-gvcsd
pl----------------------------------------------------------------------
-------------------------hetrrlcvhngh-rwd-sldlSGHtfllafsmlviw--eelrphfel
pferataaal-ateas---ss----------------------------sps---essspnlnlnlnpng--
-astsae--t--gtgrlstpalw--vlvalfvlwafmlcttal--yfHTWVEklvglllaqaiwflqyptf-
--lqth------ryp---gp--pphi--------------------------------------

>Ophiophagus_FITM1/46-290
----rrayhlwla---sv--vifgpll---------q-fy--------------------------------
---------------------------------------------------vn----pra-ifan-----hh
nffni-kfvrsa-wgwtciflggfilpvvylath-qi-----------------------------------
-------------------------lltfrhlarlavgag--lwlgate---af-----llienlt-gycfd
pvpegil-----------------------------------------------------------------
----------------------vnslpdkwtclhkgh-kwh-gydvSDHtflltfccllmv--eemaifrry
laqghp------------------------------------------------------------------
-------------agvplrpifl--lnlplesrca------sf----sc--ltssssssgtscwhaqwftst
ttatks------wel--psp--pcagssptg-a-g----t------a-ara-prgdlap-asf-

>Latimeria_FITM1/54-293
----rcyyhlwla---fi--vicgpll---------q-fy--------------------------------
---------------------------------------------------vs----prt-ifan-----kr
nffnv-afvksa-wgwtcilvggfmllvvqfscw-ri-----------------------------------
-------------------------lttlrhltrlvvgaa--lcfactq---if-----ylvedvt-gscfq
plpdgll-----------------------------------------------------------------
----------------------ltnftsklsclaEGH-lwr-gydisrptflltycsllml--eelsvfrry
lalgrl------------------------------------------------------------------
-------------asaplriifl--lncfllglwnflllctvv--yfHDYSHkvvgaaaakacwhltynww-
--ywar------wsp---gr--pgqalfpkt-v-n----t------v-kle-------------

>Human_FITM1/53-292
----rrlyhaWla---av--vifgpll---------q-fh--------------------------------
---------------------------------------------------vn----prt-ifas-----hg
nffni-kfvnsa-WgWtctflggfvllvvflatr-rv-----------------------------------
-------------------------avtarhlsrlvvgaa--vWrgagr---af-----lliedlt-gscfe
plpqgll-----------------------------------------------------------------
----------------------lhelpdrrsclaagh-qWr-gytvSSHtflltfccllma--eeaavfaky
lahglp------------------------------------------------------------------
-------------agaplrlvfl--lnvlllglWnflllctvi--yfHqytHkvvgaavgtfaWyltygsW-
--yhqp------Wsp---gs--pghglfprp-h-s----s------r-khn-------------

>Tetranychus_XP_015788491/51-346
----rkrlifylv----m--ivvggllg----dylpn-fv--------------------------------
---------------------------------------------------st----ivp-fqsn-----kk
gilnq-wfvkig-wfwtivllgpfihmtsqisaasdap---hk----sevh----qrslrddtspssrsshl
knyrdcnssl------iikl---wltlsnkdllrlwintl--fwyists---af-----twfenat-tscts
sd----------------------------------------------------------------------
-------------------------fttedacaksgh-kwi-gfdisghtfillfsllvim--eecsvmkgw
epfgerlfia-qqnrq---ktmr--------q-------------------d--------------ypq---
-----hl--iyakykifircffl--altaltliwnfmlfqtil--fyhttlqkiiayfwavglwffgykll-
--yksk------yfs---sv--fkvenlphi-k-e-----------------------------

>D_rerio_FITM2/23-252
----riylphlff---ci--slvgsvlk--------n-ae--------------------------------
---------------------------------------------------lv----pes-yfss-----sr
nvlnl-yfvkvs-wgwtivlllpfiaysnfy-iksh------------------------------------
-------------------------mfalrrltsllvatl--vwyicte---tf-----fyiedit-gscye
sntmvvi-----------------------------------------------------------------
----------------------rgefdtkaacrkagf-fwd-gfdisghsfilsysslvim--eemvpmlhi
qpay--------------------------------------------------------------------
-------------rnppldclyl--alnvivaiwiwmfgctsv--yfhdiidkilgtscgilgwymtykvw-
--yvkl------fsp---gl--ppqpkqht----------------------------------
```

```
>Latimeria_FITM2/22-261
----rknfqwvlv---ai--tvagslik--------e-vq--------------------------------
---------------------------------------------------ll----pds-yfny-----kr
nvlnv-yfvkla-wgwtlcillpfialsnfc-vsrdi-----------------------------------
-------------------------kivlkrsstlvvgta--vwyaftw---lf-----fyiedlt-gscfe
satmtav-----------------------------------------------------------------
----------------------kseygnraacrksgy-fwh-gfdiSGHsfllsycalmif--eeiavtkev
kqmksn------------------------------------------------------------------
---------ihplmnniinvlfi--alnflviiwvwmfictsv--yfHDPLHkilgtasgvlswyttykqw-
--ylkp------fsp---gl--ppkirnlke-k-v-----------------------------

>Ophiophagus_FITM2/21-261
----RRmmpwalm---sf--mvigslvk--------e-lm--------------------------------
---------------------------------------------------pp----aat-ylsn-----kr
nvlnv-yfvkfa-wawtfslllpfisltnys-vlqni-----------------------------------
-------------------------lpvlvrlfsllvgti--iwytcts---tf-----lliqdft-gscyk
sstlavv-----------------------------------------------------------------
----------------------tqehsnqlqclqagg-iwq-sfdiSGHsfllsycvlmil--eemavmpsv
ktfRGS------------------------------------------------------------------
------------RLHRvvnslfl--alacltfiwffmllttav--yfHDFWDkifgtlvglsawygtyrfw-
--ylsp------lsp---gl--ppqrtlyfp-k-p-----------n-RRl-------------

>Human_FITM2/21-262
----RRylpwalv---as--mlagsllk--------e-ls--------------------------------
---------------------------------------------------pl----pes-ylsn-----kr
nvlnv-yfvkva-wawtfclllpfialtnyh-ltgka-----------------------------------
-------------------------glvlRRlstllvgta--iwyicts---if-----sniehyt-gscyq
spalegv-----------------------------------------------------------------
----------------------rkehqskqqchqegg-fwh-gfdisghsflltfcalmiv--eemsvlhev
ktdrsh------------------------------------------------------------------
------------clhtaittlvv--algiltfiwvlmflctav--yfhnlsqkvfgtlfgllswygtygfw-
--ypka------fsp---gl--ppqscslnl-k-q-----------d-syk-k-----------

>Branchiostoma_EEN49991/29-307
----rlvlmwiv--------vivcslvh--------q-fa--------------------------------
---------------------------------------------------pl----kds-ylnn-----sd
nvlna-yfaews-wawtviptaifvlltsyvytlgev-----------------------------------
-------------------------ktvlkhqvrmvvgtm--vwgvwvg---lf-----lgvrqwt-ascsd
rs----------------------------------------------------------------------
------------------------fdtnstgceeagh-rwe-gydiSGHtfllsfcvffit--seltvllqw
drvpelleka-gtRRl---hdqq--------ssd---------------rad---epsqdgthireyqaa--
-ssssle--RFYRltpfvwplfc--llvvltflwmfllvital--yfHSVGEkliglfcgtlswwltyevw-
--yRRa------afp---gr--pgagrlgmd-g-t----p------p-eqi-e---l-------

>Branchiostoma_EEN49992/31-297
----rivvywfv--------liplsilh--------d-li--------------------------------
---------------------------------------------------pl----pes-yfsy-----kg
nvlnq-yfvkls-wawtllvttlfvsltsyvytltdt-----------------------------------
-------------------------savlkhlsrllvgtt--iwfvfts---vf-----dtvlhyt-gsctd
ae----------------------------------------------------------------------
------------------------yt-dkracvkagf-awd-gfdiSGHafllsycallit--selsvlktw
dtiqetleee-annee---rskk--------k--------------------------kgnkrkiekpde--
-retsle--wfyrltpaisvlfg--vlcsltllwefmlmttai--yfHTISHkliglacgtlswyytygrw-
--ykh-------afp---gl--pgegplqkg-g-k----R------D-Rhi-l-----------

>Nannochloropsis_EKU21300/26-339
psspvaisgfylft--lelltialvfsk-alhhnagkkyvrvvqpgchvawadmngdhst----vaccslps
ssttflsfllggddppsegqdgwk--------------nplcaaats-------------------------
pr-tk-hltsglalvlpfvtwflnclfsa---va--gwwgeggrtekgrgkd---------eegkegRRR--
-------------------------------------Rea--iwtgqRRRlglyaavimfrtavllmllnrv
qaw-----------------------------------------------------------------vq--
--------------a-------eegdtcwysalrhdg-RCRERfdfadhgvlfisqylaiqt-fetvailRE
ARs--llh----------------------------------------------------------------
---------------lvt---clasaclis-llslvgfydtvt--yfHTRTEslmgvlislvtvqlplmlva
sgRLRQ------HR--------Y----LRishf-ika---------------------------

>Albugo_FITM/24-312
aalqvkrrvwyln---vilf--slilyt---syylpykyvr-vqgrcesnwiqlnkdgsasqqgticcsddt
asisp------------------------------------cyrgme-------------------------
ls-ki-avsvkgawvfpflpliinyisvi---------lgpkpsl---------------------------
---------------------------------------e--hirvltrrallyagimlfrlmvlykllngv
ekrivpf-------------------------------------------il----------------pn--
--------------h-------dakkscwyrflrhdq-kcvdafdfSDHlillvthyiaipl-fewfalaie
```

```
spr--lwy----------------------------------------------------------------
---------------nnlrivvlrlsvfmelitavyfiyittk--yfHTPLEnvialvlteicvlyplylls
qdrlan------fvtk--rqlsw----lqlqwf-vsp--p--------ss--fk----------

>Vitrella_CEL93216/6-312
psfrtvllrwq--l--mtllafsacvyag-wRFNRgk-fs--------------------------------
----------------------ih--------------rypvaaplh-------------------------
pgeva-ffrvhpppdlpppplimeassvt---vegeeggesagisgtpaylpalpdfqrcsdegpldlsv--
----pcypfRRplqrltyspllfhlvllvppllplllcrg--fsaanrnrlllsvvvmvyravllywvplql
qefl--------------------------------------------------------------------
-----------------------------------hl-hyggsydfSDHlvlylmalmlv-a-fnq-----a
ald--cekrg--------------------------------------------------------------
-----------gg-gvvlsvvslvytml-ttavlcylgfqtvr--sfHTYEEsvgglvfglvglyfpvwila
vgmpRL------WRav--egavcgegalgRGRR-vkd-----------------------aggq

>Guillardia_XP_005839400/23-223
tgrpstqqivivyg--ipllmsflgiift-lggaadkqeg--------------------------------
----------------------vt--------------rpsapipvl-------------------------
hqfni-ifkktg-allpfvpllyRLAvsr---l-gksslpndftn--------r------------------
-----------------------h-------------ckg--ir-----ifagfvimtlira-vayslt---
------------------------------------------------------------------------
--------------------------------------llkskdimSDHvflalsvsalc-r-felhvclet
yhs--nrlih--------------------------------------------------------------
--------------------latmmnfi-ivvcvnannyvtar--yfHTTEEsvnsllcgfllfeipgtic-
----------------------------------------------------------------

>Monoraphidium_XP_013896169/57-361
lrieseaafwalls--lpllawwacvlagvwlhe-glpfg--------------------------------
--------------------------------------hkkayhlml-------------------------
wfdsa-vfrpfg-qalpfavlfariwvtt-------nwwgsggirarpaaragrqqqqqqqqqqqqqqqqqq
qqqqqqqqqqqqqqqqqqqqqqqhq--------rlqatla--wd-----clllyaaialarg-vvyllhyra
---l--------------------------------------------------------------------
-----------------------------------es-klltvhivSDHiflgatilicl-h-aeivciasd
aar--hvrrg-----------------------------------------------------------a--
-----------vtarevgltlvlvaall-ltlltaadmfftak--iyHVPEEslatavlaflvfqapvlhwl
ttrrrd-------------gavigvagvsagag-vgr--v--------ga--lagaratsgagr

>Chlamydomonas_XP_001699296/78-311
lvlpsraaltalls--lplllwagavgagclhweselpdg--------------------------------
----------------------as--------------krdfehpli-------------------------
wfdrr-vfrvwg-pllpllpflirvaaln---l-rrrpqpgggvllrpaa----------------------
-------------------------------------sla--vq-----agltygiiivvrl-llyGGHv--
---a--------------------------------------------------------------------
-----------------------------------lq-rgagvylvSDHlllaasvvacf-q-selvlcfsd
ahk--sella-------------------------------------------------------rhdpg--
-----------agaaqvavvsglvssvf-vlvalygdmyctar--wyHHPGEnlaslglgavvfqlpvlvwl
mrqarf-------------vpaa-----------------------------------------
```
